# Supplementary material for: Comprehensive Genome-Wide Natural Variation and Expression Analysis of Tubby-like Proteins Gene Family in Brachypodium distachyon
Source: Plants (Basel). 2024 Mar 29;13(7):987. doi: 10.3390/plants13070987 (PMC11013449; doi:10.3390/plants13070987)

Supplementary Figure S5. Multiple sequence alignment of all TLP proteins. Four levels of conservation are shown. F-box and Tubby domains are marked with grey and black solid lines above sequences, respectively. Blue arrows show the BdTLP protein variants identified in parental lines, orange triangles show the amino acids required for plasma membrane tethering [REFs 11, 12]

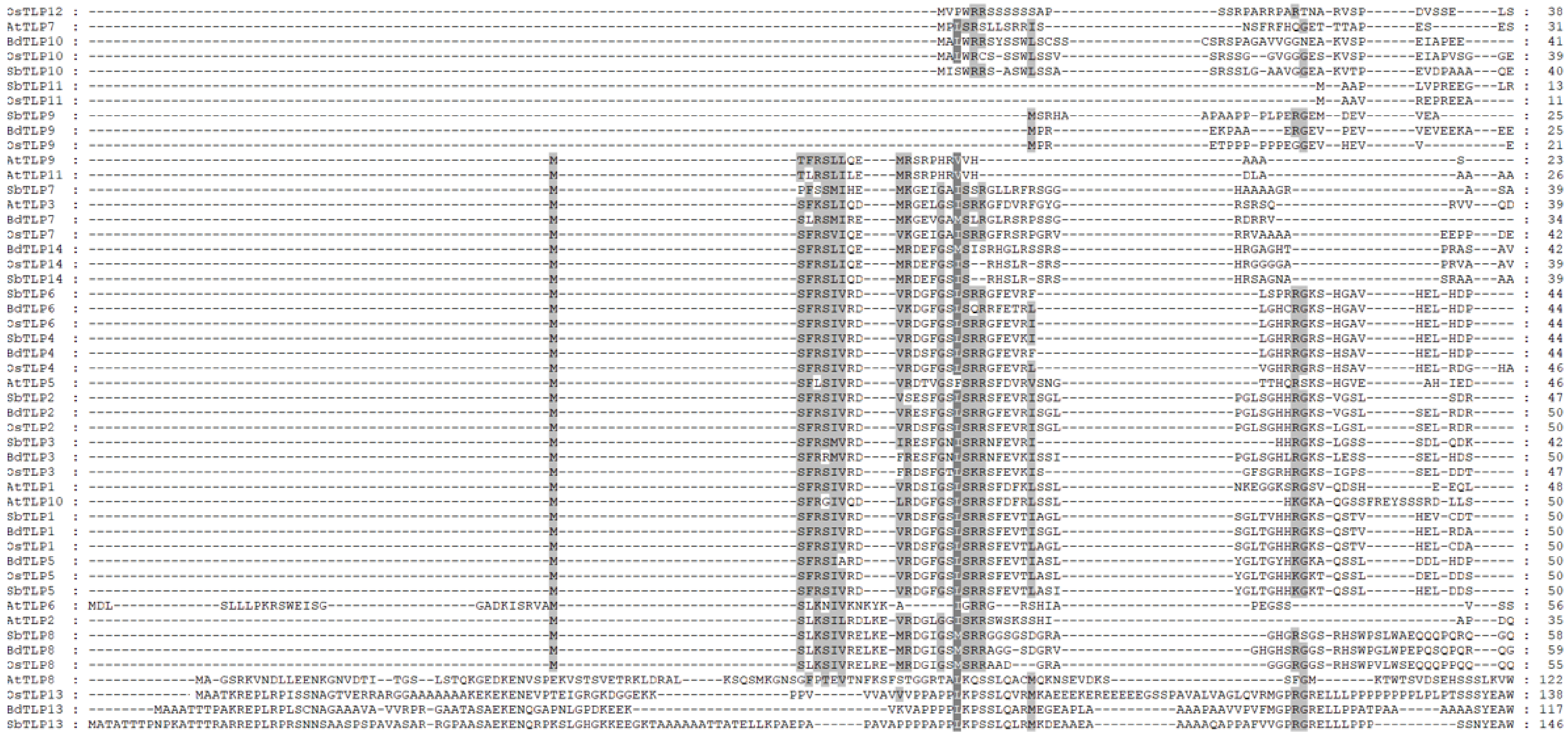

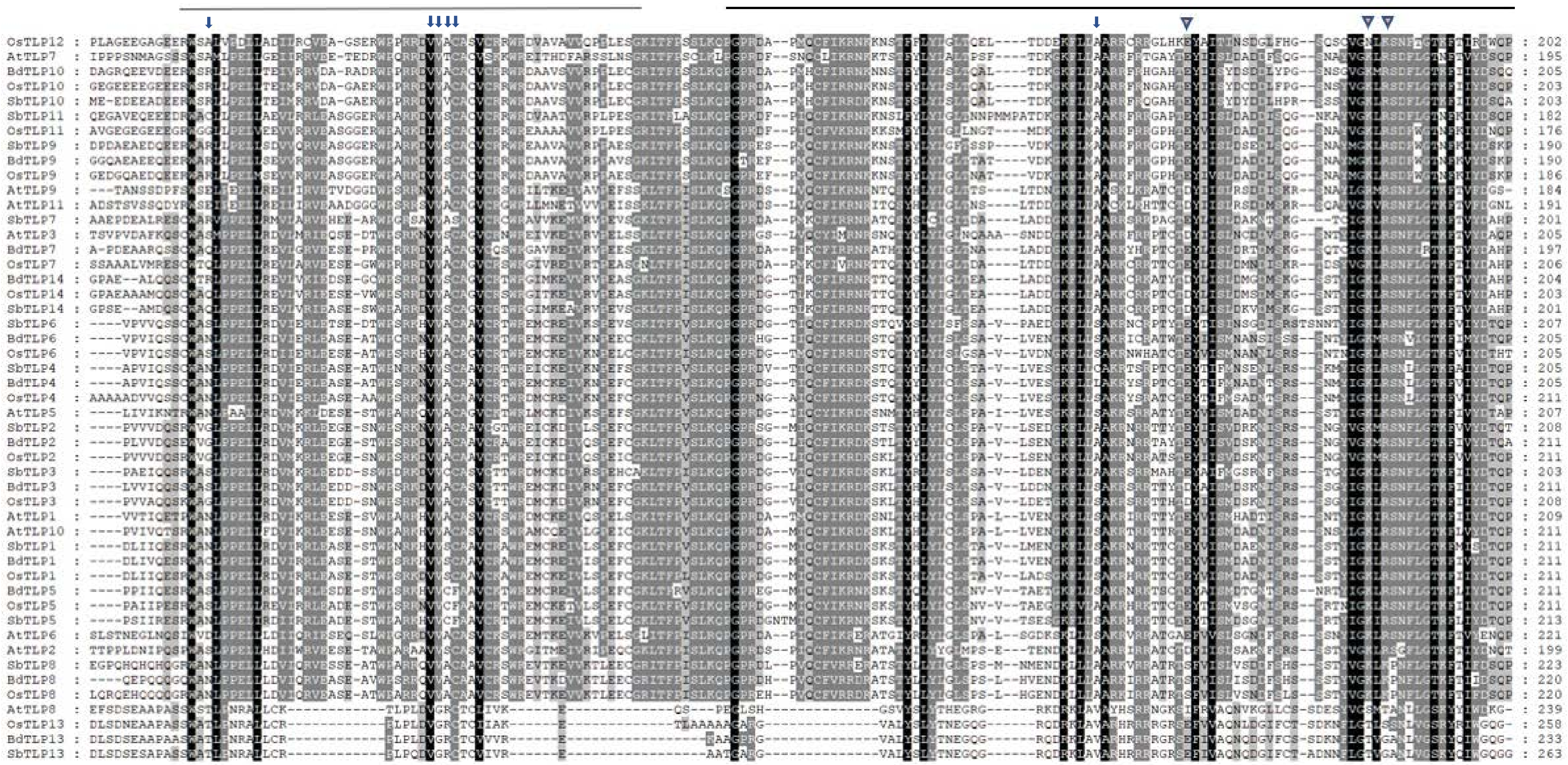

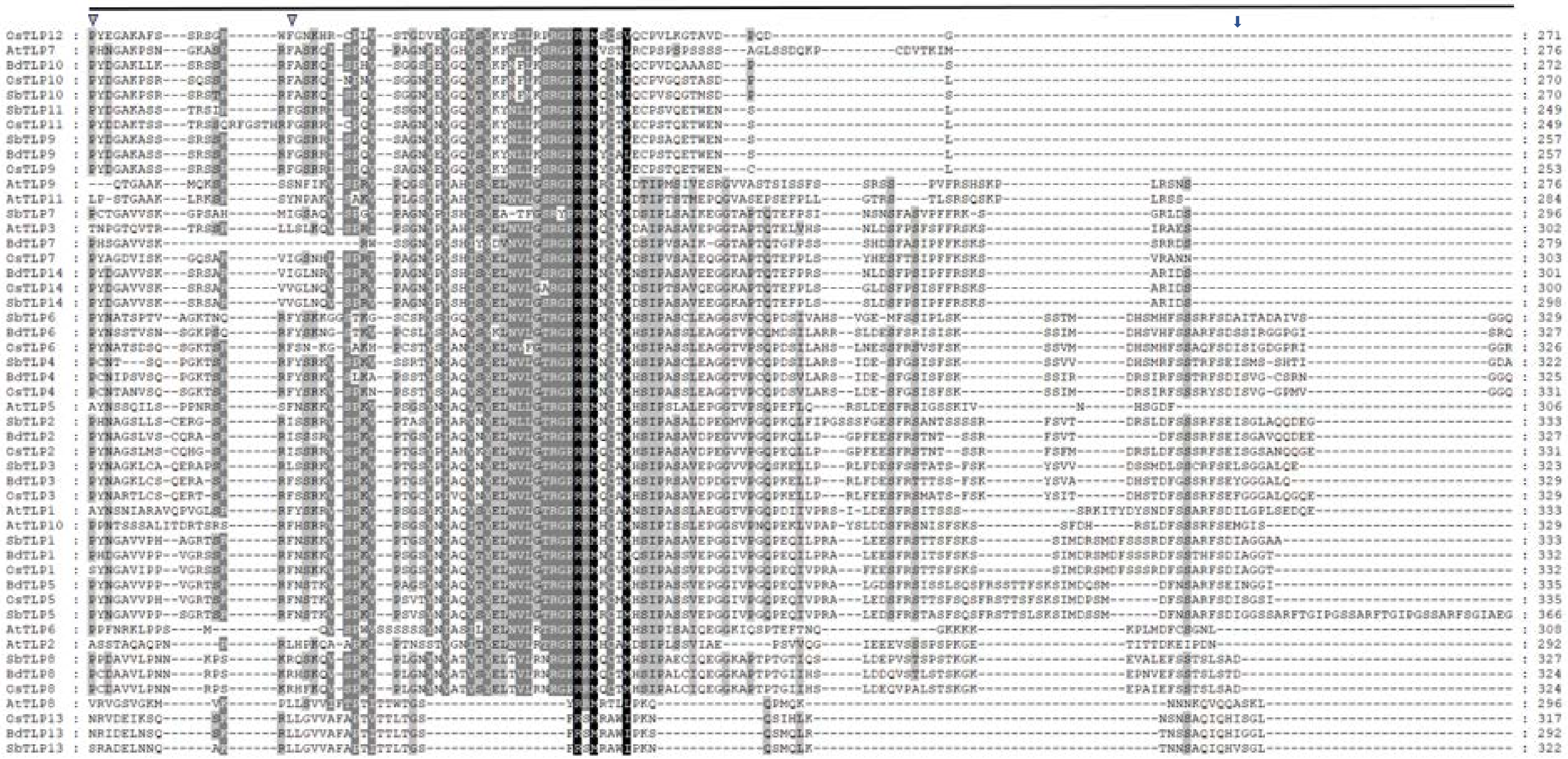

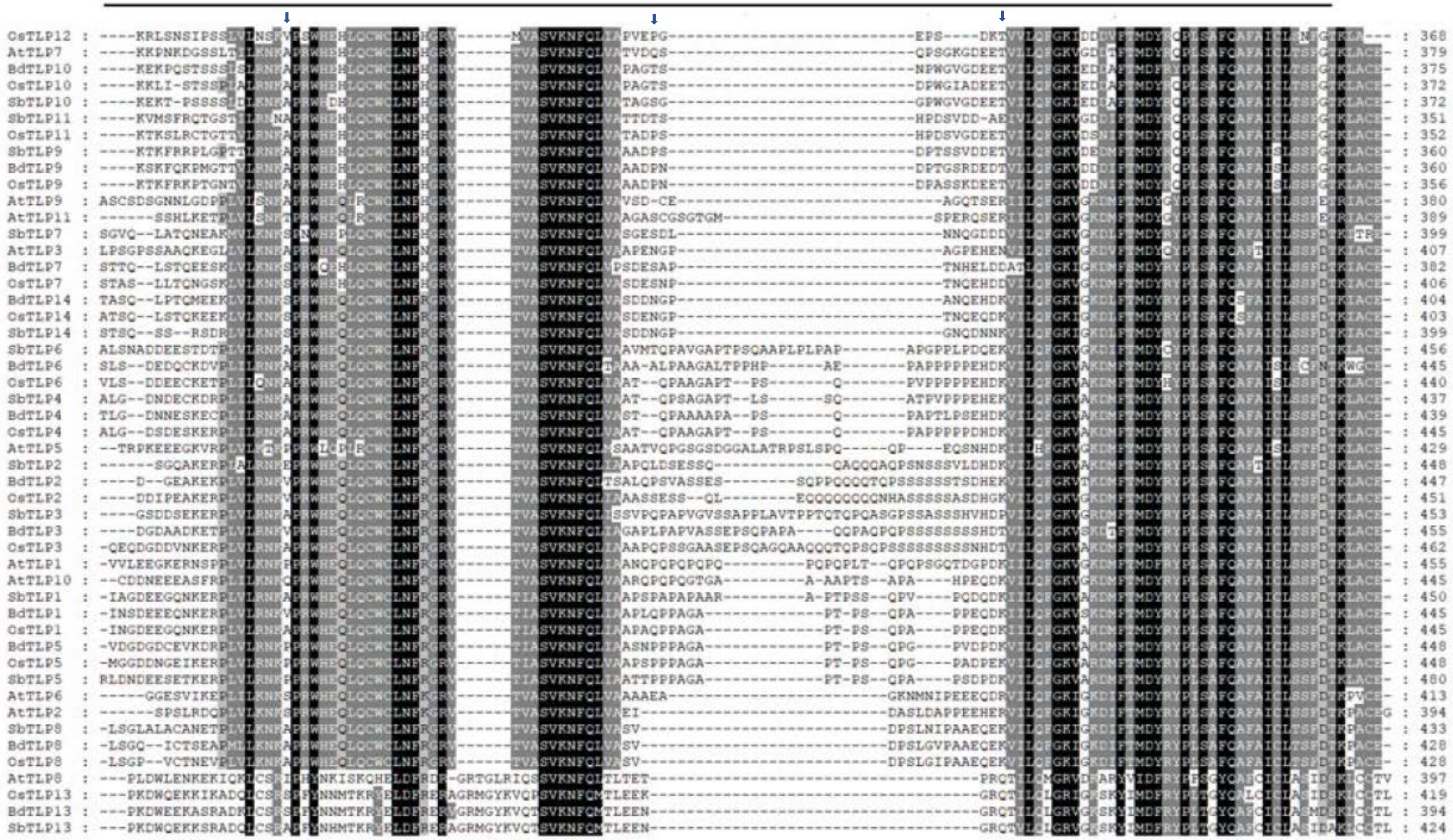

Supplement: Supplementary file 1 [file plants-13-00987-s001.zip › Figure S5. MSA of 49 BdTLP proteins.pdf]
