# Supplementary material for: Comprehensive Genome-Wide Natural Variation and Expression Analysis of Tubby-like Proteins Gene Family in Brachypodium distachyon
Source: Plants (Basel). 2024 Mar 29;13(7):987. doi: 10.3390/plants13070987 (PMC11013449; doi:10.3390/plants13070987)
Supplement: Supplementary file 1 [file plants-13-00987-s001.zip › Figure S7. MSA alignments of BdTLP in accessions.pdf]

Supplementary Figure S7. Multiple sequence alignments of eleven accessions used as parents for the generation of Recombinant Inbred Lines for all BdTLP proteins

BdTLP1

```
Bd21-3     MSFRSIVRDVRDGFGSLSRRSFEGTISGLSGLTGHHRGKSQSTVHELRDADLIVQESRWA     60
Bd3-1      MSFRSIVRDVRDGFGSLSRRSFEVTISGLSGLTGHHRGKSQSTVHELRDADLIVQESRWA     60
Bd21       MSFRSIVRDVRDGFGSLSRRSFEVTISGLSGLTGHHRGKSQSTVHELRDADLIVQESRWA     60
ABR6       MSFRSIVRDVRDGFGSLSRRSFEVTISGLSGLTGHHRGKSQSTVHELRDADLIVQESRWA     60
Bd30-1     MSFRSIVRDVRDGFGSLSRRSFEVTISGLSGLTGHHRGKSQSTVHELRDADLIVQESRWA     60
Bd2-3      MSFRSIVRDVRDGFGSLSRRSFEVTISGLSGLTGHHRGKSQSTVHELRDADLIVQESRWA     60
RON2       MSFRSIVRDVRDGFGSLSRRSFEVTISGLSGLTGHHRGKSQSTVHELRDADLIVQESRWA     60
Tek-4      MSFRSIVRDVRDGFGSLSRRSFEVTISGLSGLTGHHRGKSQSTVHELRDADLIVQESRWA     60
Bd1-1      MSFRSIVRDVRDGFGSLSRRSFEVTISGLSGLTGHHRGKSQSTVHELRDADLIVQESRWA     60
Bd29-1     MSFRSIVRDVRDGFGSLSRRSFEVTISGLSGLTGHHKGKSQSTVHELRDADLIVQESRWA     60
Koz-3      MSFRSIVRDVRDGFGSLSRRSFEVTISGLSGLTGHHKGKSQSTVHELRDADLIVQESRWA     60
           *********************** ************:***********************

Bd21-3     CLPPELLRDVIRRLEASEGTWPSRKHVVSCAAVCRAWREMCREIVISPEFCGKLTFPVSL     120
Bd3-1      CLPPELLRDVIRRLEASESTWPSRKHVVSCAAVCRAWREMCREIVISPEFCGKLTFPVSL     120
Bd21       CLPPELLRDVIRRLEASESTWPSRKHVVSCAAVCRAWREMCREIVISPEFCGKLTFPVSL     120
ABR6       CLPPELLRDVIRRLEASESTWPSRKHVVSCAAVCRAWREMCREIVISPEFCGKLTFPVSL     120
Bd30-1     CLPPELLRDVIRRLEASESTWPSRKHVVSCAAVCRAWREMCREIVISPEFCGKLTFPVSL     120
Bd2-3      CLPPELLRDVIRRLEASESTWPSRKHVVSCAAVCRAWREMCREIVISPEFCGKLTFPVSL     120
RON2       CLPPELLRDVIRRLEASESTWPSRKHVVSCAAVCRAWREMCREIVISPEFCGKLTFPVSL     120
Tek-4      SLPPELLRDVIRRLEASESTWPSRKHVVSCAAVCRAWREMCREIVISPEFCGKLTFPVSL     120
Bd1-1      SLPPELLRDVIRRLEASESTWPSRKHVVSCAAVCRAWREMCREIVISPEFCGKLTFPVSL     120
Bd29-1     SLPPELLRDVIRRLEASESTWPSRKHVVSCAAVCRAWREMCREIVISPEFCGKLTFPVSL     120
Koz-3      SLPPELLRDVIRRLEASESTWPSRKHVVSCAAVCRAWREMCREIVISPEFCGKLTFPVSL     120
           .*****************.*****************************************

Bd21-3     KQPGPRDGMIQCFIKRDKSKSTYHLYLCLSTAVLVENGKFLLSAKRNRKTTCTEYVISMD     180
Bd3-1      KQPGPRDGMIQCFIKRDKSKSTYHLYLCLSTAVLVENGKFLLSAKRNRKTTCTEYVISMD     180
Bd21       KQPGPRDGMIQCFIKRDKSKSTYHLYLCLSTAVLVENGKFLLSAKRNRKTTCTEYVISMD     180
ABR6       KQPGPRDGMIQCFIKRDKSKSTYHLYLCLSTAVLVENGKFLLSAKRNRKTTCTEYVISMD     180
Bd30-1     KQPGPRDGMIQCFIKRDKSKSTYHLYLCLSTAVLVENGKFLLSAKRNRKTTCTEYVISMD     180
Bd2-3      KQPGPRDGMIQCFIKRDKSKSTYHLYLCLSTAVLVENGKFLLSAKRNRKTTCTEYVISMD     180
RON2       KQPGPRDGMIQCFIKRDKSKSTYHLYLCLSTAVLVENGKFLLSAKRNRKTTCTEYVISMD     180
Tek-4      KQPGPRDGMIQCFIKRDKSKSTYHLYLCLSTAVLVENGKFLLSAKRNRKTTCTEYVISMD     180
Bd1-1      KQPGPRDGMIQCFIKRDKSKSTYHLYLCLSTAVLVENGKFLLSAKRNRKTTCTEYVISMD     180
Bd29-1     KQPGPRDGMIQCFIKRDKSKSTYHLYLCLSTAVLVENGKFLLSAKRNRKTTCTEYVISMD     180
Koz-3      KQPGPRDGMIQCFIKRDKSKSTYHLYLCLSTAVLVENGKFLLSAKRNRKTTCTEYVISMD     180
           ************************************************************

Bd21-3     ADNISRSSSTYIGKLRSNFLGTKFIVYDTQPPHDGAVVPPVGRSSRRFNSKKVSPKVPSG     240
Bd3-1      ADNISRSSSTYIGKLRSNFLGTKFIVYDTQPPHDGAVVPPVGRSSRRFNSKKVSPKVPSG     240
Bd21       ADNISRSSSTYIGKLRSNFLGTKFIVYDTQPPHDGAVVPPVGRSSRRFNSKKVSPKVPSG     240
ABR6       ADNISRSSSTYIGKLRSNFLGTKFIVYDTQPPHDGAVVPPVGRSSRRFNSKKVSPKVPSG     240
Bd30-1     ADNISRSSSTYIGKLRSNFLGTKFIVYDTQPPHDGAVVPPVGRSSRRFNSKKVSPKVPSG     240
Bd2-3      ADNISRSSSTYIGKLRSNFLGTKFIVYDTQPPHDGAVVPPVGRSSRRFNSKKVSPKVPSG     240
RON2       ADNISRSSSTYIGKLRSNFLGTKFIVYDTQPPHDGAVVPPVGRSSRRFNSKKVSPKVPSG     240
Tek-4      ADNISRSSSTYIGKLRSNFLGTKFIVYDTQPPHDGAVVPPVGRSSRRFNSKKVSPKVPSG     240
Bd1-1      ADNISRSSSTYIGKLRSNFLGTKFIVYDTQPPHDGAVVPPVGRSSRRFNSKKVSPKVPSG     240
Bd29-1     ADNISRSSSTYIGKLRSNFLGTKFIVYDTQPPHDGAVVPPVGRSSRRFNSKKVSPKVPSG     240
Koz-3      ADNISRSSSTYIGKLRSNFLGTKFIVYDTQPPHDGAVVPPVGRSSRRFNSKKVSPKVPSG     240
           ************************************************************
```

```
Bd21-3      SYNIAQVTYELNVLGTRGPRRMNCIMQSIPASSVEPGGIVPGQPEQILPRALEESFRSIT      300
Bd3-1       SYNIAQVTYELNVLGTRGPRRMNCIMQSIPASSVEPGGIVPGQPEQILPRALEESFRSIT      300
Bd21        SYNIAQVTYELNVLGTRGPRRMNCIMQSIPASSVEPGGIVPGQPEQILPRALEESFRSIT      300
ABR6        SYNIAQVTYELNVLGTRGPRRMNCIMQSIPASSVEPGGIVPGQPEQILPRALEESFRSIT      300
Bd30-1      SYNIAQVTYELNVLGTRGPRRMNCIMQSIPASSVEPGGIVPGQPEQILPRALEESFRSIT      300
Bd2-3       SYNIAQVTYELNVLGTRGPRRMNCIMQSIPASSVEPGGIVPGQPEQILPRALEESFRSIT      300
RON2        SYNIAQVTYELNVLGTRGPRRMNCIMQSIPASSVEPGGIVPGQPEQILPRALEESFRSIT      300
Tek-4       SYNIAQVTYELNVLGTRGPRRMNCIMQSIPASSVEPGGIVPGQPEQILPRALEESFRSIT      300
Bd1-1       SYNIAQVTYELNVLGTRGPRRMNCIMQSIPASSVEPGGIVPGQPEQILPRALEESFRSIT      300
Bd29-1      SYNIAQVTYELNVLGTRGPRRMNCIMQSIPASSVEPGGIVPGQPEQILPRALEESFRSIT      300
Koz-3       SYNIAQVTYELNVLGTRGPRRMNCIMQSIPASSVEPGGIVPGQPEQILPRALEESFRSIT      300
            ************************************************************

Bd21-3      SFSKSSIMDRSMDFSSSRVFSSTHFSDIAGGTINSDEEEQNKERPLVLRNKVPRWHEQLQ      360
Bd3-1       SFSKSSIMDRSMDFSSSRDFSSTHFSDIAGGTINSDEEEQNKERPLVLRNKVPRWHEQLQ      360
Bd21        SFSKSSIMDRSMDFSSSRDFSSTHFSDIAGGTINSDEEEQNKERPLVLRNKVPRWHEQLQ      360
ABR6        SFSKSSIMDRSMDFSSSRDFSSTHFSDIAGGTINSDEEEQNKERPLVLRNKVPRWHEQLQ      360
Bd30-1      SFSKSSIMDRSMDFSSSRDFSSTHFSDIAGGTINSDEEEQNKERPLVLRNKVPRWHEQLQ      360
Bd2-3       SFSKSSIMDRSMDFSSSRDFSSTHFSDIAGGTINSDEEEQNKERPLVLRNKVPRWHEQLQ      360
RON2        SFSKSSIMDRSMDFSSSRDFSSTHFSDIAGGTINSDEEEQNKERPLVLRNKVPRWHEQLQ      360
Tek-4       SFSKSSIMDRSMDFSSSRDFSSTHFSDIAGGTINSDDEEQNKERPLVLRNKVPRWHEQLQ      360
Bd1-1       SFSKSSIMDRSMDFSSSRDFSSTHFSDIAGGTINSDDEEQNKERPLVLRNKVPRWHEQLQ      360
Bd29-1      SFSKSSIMDQSMDFSSSRDFSSTHFSDIAGGTINSDEEEQNKERPLVLRNKVPRWHEQLQ      360
Koz-3       SFSKSSIMDQSMDFSSSRDFSSTHFSDIAGGTINSDEEEQNKERPLVLRNKVPRWHEQLQ      360
            *********:******** *****************:***********************

Bd21-3      CWCLNFRGRVTIASVKNFQLIAAPLQPPAGAPTPSQPAPPEQDKIILQFGKVSKDMFTMD      420
Bd3-1       CWCLNFRGRVTIASVKNFQLIAAPLQPPAGAPTPSQPAPPEQDKIILQFGKVSKDMFTMD      420
Bd21        CWCLNFRGRVTIASVKNFQLIAAPLQPPAGAPTPSQPAPPEQDKIILQFGKVSKDMFTMD      420
ABR6        CWCLNFRGRVTIASVKNFQLIAAPLQPPAGAPTPSQPAPPEQDKIILQFGKVSKDMFTMD      420
Bd30-1      CWCLNFRGRVTIASVKNFQLIAAPLQPPAGAPTPSQPAPPEQDKIILQFGKVSKDMFTMD      420
Bd2-3       CWCLNFRGRVTIASVKNFQLIAAPLQPPAGAPTPSQPAPPEQDKIILQFGKVSKDMFTMD      420
RON2        CWCLNFRGRVTIASVKNFQLIAAPLQPPAGAPTPSQPAPPEQDKIILQFGKVSKDMFTMD      420
Tek-4       CWCLNFRGRVTIASVKNFQLIAAPLQPPAGAPTPSQPAPPEQDKIILQFGKVSKDMFTMD      420
Bd1-1       CWCLNFRGRVTIASVKNFQLIAAPLQPPAGAPTPSQPAPPEQDKIILQFGKVSKDMFTMD      420
Bd29-1      CWCLNFRGRVTIASVKNFQLIAAPLQPPAGAPTPSQPAPPEQDKIILQFGKVSKDMFTMD      420
Koz-3       CWCLNFRGRVTIASVKNFQLIAAPLQPPAGAPTPSQPAPPEQDKIILQFGKVSKDMFTMD      420
            ************************************************************

Bd21-3      YRYPLSAFQAFAICLSSFDTKLACE   445
Bd3-1       YRYPLSAFQAFAICLSSFDTKLACE   445
Bd21        YRYPLSAFQAFAICLSSFDTKLACE   445
ABR6        YRYPLSAFQAFAICLSSFDTKLACE   445
Bd30-1      YRYPLSAFQAFAICLSSFDTKLACE   445
Bd2-3       YRYPLSAFQAFAICLSSFDTKLACE   445
RON2        YRYPLSAFQAFAICLSSFDTKLACE   445
Tek-4       YRYPLSAFQAFAICLSSFDTKLACE   445
Bd1-1       YRYPLSAFQAFAICLSSFDTKLACE   445
Bd29-1      YRYPLSAFQAFAICLSSFDTKLACE   445
Koz-3       YRYPLSAFQAFAICLSSFDTKLACE   445
            *************************
```

BdTLP2

```
Bd21-3      MSFRSIVRDVRESFGSLSRRGFEVRISGLPGLSGHHRGKSVGSLSELRDRPLVVDQSEWV    60
Tek-4       MSFRSIVRDVRESFGSLSRRGFEVRISGLPGLSGHHRGKSVGSLSELRDRPLVVDQSEWV    60
Bd3-1       MSFRSIVRDVRESFGSLSRRGFEVRISGLPGLSGHHRGKSVGSLSELRDRPLVVDQSEWV    60
Bd21        MSFRSIVRDVRESFGSLSRRGFEVRISGLPGLSGHHRGKSVGSLSELRDRPLVVDQSEWV    60
ABR6        MSFRSIVRDVRESFGSLSRRGFEVRISGLPGLSGHHRGKSVGSLSELRDRPLVVDQSEWV    60
Bd30-1      MSFRSIVRDVRESFGSLSRRGFEVRISGLPGLSGHHRGKSVGSLSELRDRPLVVDQSEWV    60
Bd2-3       MSFRSIVRDVRESFGSLSRRGFEVRISGLPGLSGHHRGKSVGSLSELRDRPLVVDQSEWV    60
RON2        MSFRSIVRDVRESFGSLSRRGFEVRISGLPGLSGHHRGKSVGSLSELRDRPLVVDQSEWV    60
Koz-3       MSFRSIVRDVRESFGSLSRRGFEVRISGLPGLSGHHRGKSVGSLSELRDRPLVVDQSEWV    60
Bd29-1      MSFRSIVRDVRESFGSLSRRGFEVRISGLPGLSGHHRGKSVGSLSELRDRPLVVDQSEWV    60
Bd1-1       MSFRSIVRDVRESFGSLSRRGFEVRISGLPGLSGHHRGKSVGSLSELRDRPLVVDQSEWV    60
            ************************************************************

Bd21-3      GLPPELLRDVMKRLEEGESTWPSRKDVVACAAVCRAWREICKDIVQSPEFCGKLTFPVSL    120
Tek-4       GLPPELLRDVMKRLEEGESTWPSRKDVVACAAVCRAWREICKDIVQSPEFCGKLTFPVSL    120
Bd3-1       GLPPELLRDVMKRLEEGESTWPSRKDVVACAAVCRAWREICKDIVQSPEFCGKLTFPVSL    120
Bd21        GLPPELLRDVMKRLEEGESTWPSRKDVVACAAVCRAWREICKDIVQSPEFCGKLTFPVSL    120
ABR6        GLPPELLRDVMKRLEEGESTWPSRKDVVACAAVCRAWREICKDIVQSPEFCGKLTFPVSL    120
Bd30-1      GLPPELLRDVMKRLEEGESTWPSRKDVVACAAVCRAWREICKDIVQSPEFCGKLTFPVSL    120
Bd2-3       GLPPELLRDVMKRLEEGESTWPSRKDVVACAAVCRAWREICKDIVQSPEFCGKLTFPVSL    120
RON2        GLPPELLRDVMKRLEEGESTWPSRKDVVACAAVCRAWREICKDIVQSPEFCGKLTFPVSL    120
Koz-3       GLPPELLRDVMKRLEEGESTWPSRKDVVACAAVCRAWREICKDIVQSPEFCGKLTFPVSL    120
Bd29-1      GLPPELLRDVMKRLEEGESTWPSRKDVVACAAVCRAWREICKDIVQSPEFCGKLTFPVSL    120
Bd1-1       GLPPELLRDVMKRLEEGESTWPSRKDVVACAAVCRAWREICKDIVQSPEFCGKLTFPVSL    120
            ************************************************************

Bd21-3      KQPGPRDGLIQCFIKRDKSTLTYYLYLCLSPAVLSENGKFLLAAKRNRRTAYTEYVISVD    180
Tek-4       KQPGPRDGLIQCFIKRDKSTLTYYLYLCLSPAVLSENGKFLLAAKRNRRTAYTEYVISVD    180
Bd3-1       KQPGPRDGLIQCFIKRDKSTLTYYLYLCLSPAVLSENGKFLLAAKRNRRTAYTEYVISVD    180
Bd21        KQPGPRDGLIQCFIKRDKSTLTYYLYLCLSPAVLSENGKFLLAAKRNRRTAYTEYVISVD    180
ABR6        KQPGPRDGLIQCFIKRDKSTLTYYLYLCLSPAVLSENGKFLLAAKRNRRTAYTEYVISVD    180
Bd30-1      KQPGPRDGLIQCFIKRDKSTLTYYLYLCLSPAVLSENGKFLLAAKRNRRTAYTEYVISVD    180
Bd2-3       KQPGPRDGLIQCFIKRDKSTLTYYLYLCLSPAVLSENGKFLLAAKRNRRTAYTEYVISVD    180
RON2        KQPGPRDGLIQCFIKRDKSTLTYYLYLCLSPAVLSENGKFLLAAKRNRRTAYTEYVISVD    180
Koz-3       KQPGPRDGLIQCFIKRDKSTLTYYLYLCLSPAVLSENGKFLLAAKRNRRTAYTEYVISVD    180
Bd29-1      KQPGPRDGLIQCFIKRDKSTLTYYLYLCLSPAVLSENGKFLLAAKRNRRTAYTEYVISVD    180
Bd1-1       KQPGPRDGLIQCFIKRDKSTLTYYLYLCLSPAVLSENGKFLLAAKRNRRTAYTEYVISVD    180
            ************************************************************

Bd21-3      SKNISRSSNGYVGKMRG-------------TPYNAGSLVSCQRASRRISSSRVSPKVPTG    227
Tek-4       SKNISRSSNGYVGKMRSNFLGTKFIVYDTQAPYNAGSLVSCQRASRRISSSRVSPKVPTG    240
Bd3-1       SKNISRSSNGYVGKMRSNFLGTKFIVYDTQAPYNAGSLVSCQRASRRISSSRVSPKVPTG    240
Bd21        SKNISRSSNGYVGKMRSNFLGTKFIVYDTQAPYNAGSLVSCQRASRRISSSRVSPKVPTG    240
ABR6        SKNISRSSNGYVGKMRSNFLGTKFIVYDTQAPYNAGSLVSCQRASRRISSSRVSPKVPTG    240
Bd30-1      SKNISRSSNGYVGKMRSNFLGTKFIVYDTQAPYNAGSLVSCQRASRRISSSRVSPKVPTG    240
Bd2-3       SKNISRSSNGYVGKMRSNFLGTKFIVYDTQAPYNAGSLVSCQRASRRISSSRVSPKVPTG    240
RON2        SKNISRSSNGYVGKMRSNFLGTKFIVYDTQAPYNAGSLVSCQRASRRISSSRVSPKVPTG    240
Koz-3       SKNISRSSNGYVGKMRSNFLGTKFIVYDTQAPYNAGSLVSCQRASRRISSSRVSPKVPTG    240
Bd29-1      SKNISRSSNGYVGKMRSNFLGTKFIVYDTQAPYNAGSLVSCQRASRRISSSRVSPKVPTG    240
Bd1-1       SKNISRSSNGYVGKMRSNFLGTKFIVYDTQAPYNAGSLVSCQRASRRISSSRVSPKVPTG    240
            ****************.             :*****************************
```

```
Bd21-3      SYPIAQVNYELNVLGTRGPRRMQCTMHSIPASAVDPEGVVPGQPKQLLPGPFEESFRSTN       287
Tek-4       SYPIAQVNYELNVLGTRGPRRMQCTMHSIPASAVDPEGVVPGQPKQLLPGPFEE------       294
Bd3-1       SYPIAQVNYELNVLGTRGPRRMQCTMHSIPASAVDPEGVVPGQPKQLLPGPFEESFRSTN       300
Bd21        SYPIAQVNYELNVLGTRGPRRMQCTMHSIPASAVDPEGVVPGQPKQLLPGPFEESFRSTN       300
ABR6        SYPIAQVNYELNVLGTRGPRRMQCTMHSIPASAVDPEGVVPGQPKQLLPGPFEESFRSTN       300
Bd30-1      SYPIAQVNYELNVLGTRGPRRMQCTMHSIPASAVDPEGVVPGQPKQLLPGPFEESFRSTN       300
Bd2-3       SYPIAQVNYELNVLGTRGPRRMQCTMHSIPASAVDPEGVVPGQPKQLLPGPFEESFRSTN       300
RON2        SYPIAQVNYELNVLGTRGPRRMQCTMHSIPASAVDPEGVVPGQPKQLLPGPFEESFRSTN       300
Koz-3       SYPIAQVNYELNVLGTRGPRRMQCTMHSIPASAVDPEGVVPGQPKQLLPGPFEESFRSTN       300
Bd29-1      SYPIAQVNYELNVLGTRGPRRMQCTMHSIPASAVDPEGVVPGQPKQLLPGPFEESFRSTN       300
Bd1-1       SYPIAQVNYELNVLGTRGPRRMQCTMHSIPASAVDPEGVVPGQPKQLLPGPFEESFRSTN       300
            ******************************************************

Bd21-3      TSSRFSVTDFSSSRFSEISGAVQQDEEDGEAKEKPLVLRNKVPRWHEQLQCWCLNFRGRV       347
Tek-4       ----FSVTDFSSSRFSEISGAVQQHEEDGEAKEKPLVLRNKVPRWHEQLQCWCLNFRGRV       350
Bd3-1       TSSRFSVTDFSSSRFSEISGAVQQDEEDGEAKEKPLVLRNKVPRWHEQLQCWCLNFRGRV       360
Bd21        TSSRFSVTDFSSSRFSEISGAVQQDEEDGEAKEKPLVLRNKVPRWHEQLQCWCLNFRGRV       360
ABR6        TSSRFSVTDFSSSRFSEISGAVQQDEEDGEAKEKPLVLRNKVPRWHEQLQCWCLNFRGRV       360
Bd30-1      TSSRFSVTDFSSSRFSEISGAVQQDEEDGEAKEKPLVLRNKVPRWHEQLQCWCLNFRGRV       360
Bd2-3       TSSRFSVTDFSSSRFSEISGAVQQDEEDGEAKEKPLVLRNKVPRWHEQLQCWCLNFRGRV       360
RON2        TSSRFSVTDFSSSRFSEISGAVQQDEEDGEAKEKPLVLRNKVPRWHEQLQCWCLNFRGRV       360
Koz-3       TSSRFSVTDFSSSRFSEISGAVQQDEEDGEAKEKPLVLRNKVPRWHEQLQCWCLNFRGRV       360
Bd29-1      TSSRFSVTDFSSSRFSEISGAVQQHEEDGEAKEKPLVLRNKVPRWHEQLQCWCLNFRGRV       360
Bd1-1       TSSRFSVTDFSSSRFSEISGAVQQHEEDGEAKEKPLVLRNKVPRWHEQLQCWCLNFRGRV       360
                ********************.***********************************

Bd21-3      TVASVKNFQLTSALQPSVASSESSQPPQQQQTQPSSSSSSTSDHEKVILQFGKVTKDMFT       407
Tek-4       TVASVKNFQLTSAPQPSVASSESSQPPQQQQTQPSSSSSSTSDHEKVILQFGKVTKDMFT       410
Bd3-1       TVASVKNFQLTSALQPSVASSESSQPPQQQQTQPSSSSSSTSDHEKVILQFGKVTKDMFT       420
Bd21        TVASVKNFQLTSALQPSVASSESSQPPQQQQTQPSSSSSSTSDHEKVILQFGKVTKDMFT       420
ABR6        TVASVKNFQLTSALQPSVASSESSQPPQQQQTQPSSSSSSTSDHEKVILQFGKVTKDMFT       420
Bd30-1      TVASVKNFQLTSALQPSVASSESSQPPQQQQTQPSSSSSSTSDHEKVILQFGKVTKDMFT       420
Bd2-3       TVASVKNFQLTSALQPSVASSESSQPPQQQQTQPSSSSSSTSDHEKVILQFGKVTKDMFT       420
RON2        TVASVKNFQLTSALQPSVASSESSQPPQQQQTQPSSSSSSTSDHEKVILQFGKVTKDMFT       420
Koz-3       TVASVKNFQLTSALQPSVASSESSQPPQQQQTQPSSSSSSTSDHEKVILQFGKVTKDMFT       420
Bd29-1      TVASVKNFQLTSAPQPSVASSESSQPPQQQQTQPSSSSSSTSDHEKVILQFGKVTKDMFT       420
Bd1-1       TVASVKNFQLTSAPQPSVASSESSQPPQQQQTQPSSSSSSTSDHEKVILQFGKVTKDMFT       420
            ************* **********************************************

Bd21-3      MAYRYPLSAFQAFAICLTSFDTKLACE 434
Tek-4       MDYRYPLSAFQAFAICLTSFDTKLACE 437
Bd3-1       MDYRYPLSAFQAFAICLTSFDTKLACE 447
Bd21        MDYRYPLSAFQAFAICLTSFDTKLACE 447
ABR6        MDYRYPLSAFQAFAICLTSFDTKLACE 447
Bd30-1      MDYRYPLSAFQAFAICLTSFDTKLACE 447
Bd2-3       MDYRYPLSAFQAFAICLTSFDTKLACE 447
RON2        MDYRYPLSAFQAFAICLTSFDTKLACE 447
Koz-3       MDYRYPLSAFQAFAICLTSFDTKLACE 447
Bd29-1      MDYRYPLSAFQAFAICLTSFDTKLACE 447
Bd1-1       MDYRYPLSAFQAFAICLTSFDTKLACE 447
            * *************************
```

BdTLP3

```
Koz-3     MSFRRMVRDFRESFGNLSRRNFEVKISSIPGLSGHLRGKSLESSSELHDSLVVIQQSSWA     60
Bd21-3    MSFRRMVRDFRESFGNLSRRNFEVKISSIPGLSGHLRGKSLESSSELHDSLVVIQQSSWA     60
Bd2-3     MSFRRMVRDFRESFGNLSRRNFEVKISSIPGLSGHLRGKSLESSSELHDSLVVIQQSSWA     60
Bd3-1     MSFRRMVRDFRESFGNLSRRNFEVKISSIPGLSGHLRGKSLESSSELHDSLVVIQQSSWA     60
Bd21      MSFRRMVRDFRESFGNLSRRNFEVKISSIPGLSGHLRGKSLESSSELHDSLVVIQQSSWA     60
Bd30-1    MSFRRMVRDFRESFGNLSRRNFEVKISSIPGLSGHLRGKSLESSSELHDSLVVIQQSSWA     60
Tek-4     MSFRRMVRDFRESFGNLSRRNFEVKISSIPGLSGHLRGKSLESSSELHDSLVVIQQSSWA     60
ABR6      MSFRRMVRDFRESFGNLSRRNFEVKISSIPGLSGHLRGKSLESSSELHDSLVVIQQSSWA     60
Bd29-1    MSFRRMVRDFRESFGNLSRRNFEVKISSIPGLSGHLRGKSLESSSELHDSLVVIQQSSWA     60
Bd1-1     MSFRRMVRDFRESFGNLSRRNFEVKISSIPGLSGHLRGKSLESSSELHDSLVVIQQSSWA     60
RON2      MSFRRMVRDFRESFGNLSRRNFEVKISSIPGLSGHLRGKSLESSSELHDSLVVIQQSSWA     60
          ************************************************************

Koz-3     SLPPELLRDVMKRLEEDDSNWPSRKDVVACASVCTTWREMCKDIVRNPEFCGKLTFPVSL     120
Bd21-3    SLPPELLRDVMKRLEEDDSNWPSRKDVVACASVCTTWREMCKDIVRNPEFCGKLTFPVSL     120
Bd2-3     SLPPELLRDVMKRLEEDDSNWPSRKDVVACASVCTTWREMCKDIVRNPEFCGKLTFPVSL     120
Bd3-1     SLPPELLRDVMKRLEEDDSNWPSRKDVVACASVCTTWREMCKDIVRNPEFCGKLTFPVSL     120
Bd21      SLPPELLRDVMKRLEEDDSNWPSRKDVVACASVCTTWREMCKDIVRNPEFCGKLTFPVSL     120
Bd30-1    SLPPELLRDVMKRLEEDDSNWPSRKDVVACASVCTTWREMCKDIVRNPEFCGKLTFPVSL     120
Tek-4     SLPPELLRDVMKRLEEDDSNWPSRKDVVACASVCTTWREMCKDIVRNPEFCGKLTFPVSL     120
ABR6      SLPPELLRDVMKRLEEDDSNWPSRKDVVACASVCTTWREMCKDIVRNPEFCGKLTFPVSL     120
Bd29-1    SLPPELLRDVMKRLEEDDSNWPSRKDVVACASVCTTWREMCKDIVRNPEFCGKLTFPVSL     120
Bd1-1     SLPPELLRDVMKRLEEDDSNWPSRKDVVACASVCTTWREMCKDIVRNPEFCGKLTFPVSL     120
RON2      SLPPELLRDVMKRLEEDDSNWPSRKDVVACASVCTTWREMCKDIVRNPEFCGKLTFPVSL     120
          ************************************************************

Koz-3     KQPGPRDGLIQCFIKRDKSKLTYHLYLSLTSAVLDDNGKFLLSAKRSRRTT---------     171
Bd21-3    KQPGPRDGLIQCFIKRDKSKLTYHLYLSLTSAVLDDNGKFLLSAKRSRRTT---------     171
Bd2-3     KQPGPRDGLIQCFIKRDKSKLTYHLYLSLTSAVLDDNGKFLLSAKRSRRTTYTDYAISMD     180
Bd3-1     KQPGPRDGLIQCFIKRDKSKLTYHLYLSLTSAVLDDNGKFLLSAKRSRRTTYTDYAISMD     180
Bd21      KQPGPRDGLIQCFIKRDKSKLTYHLYLSLTSAVLDDNGKFLLSAKRSRRTTYTDYAISMD     180
Bd30-1    KQPGPRDGLIQCFIKRDKSKLTYHLYLSLTSAVLDDNGKFLLSAKRSRRTTYTDYAISMD     180
Tek-4     KQPGPRDGLIQCFIKRDKSKLTYHLYLSLTSAVLDDNGKFLLSAKRSRRTTYTDYAISMD     180
ABR6      KQPGPRDGLIQCFIKRDKSKLTYHLYLSLTSAVLDDNGKFLLSAKRSRRTTYTDYAISMD     180
Bd29-1    KQPGPRDGLIQCFIKRDKSKLTYHLYLSLTSAVLDDNGKFLLSAKRSRRTTYTDYAISMD     180
Bd1-1     KQPGPRDGLIQCFIKRDKSKLTYHLYLSLTSAVLDDNGKFLLSAKRSRRTTYTDYAISMD     180
RON2      KQPGPRDGLIQCFIKRDKSKLTYHLYLSLTSAVLDDNGKFLLSAKRSRRTTYTDYAISMD     180
          ***************************************************

Koz-3     ----------------SNFLGPKFIIYDTQPPYNAGKLCSQERASRRFSSRKVSPKVPTG     215
Bd21-3    ----------------SNFLGTKFIIYDTQPPYNAGKLCSQERASRRFSSRKVSPKVPTG     215
Bd2-3     SKNISRSSSGYIGELRSNFLGTKFIIYDTQPPYNAGKLCSQERASRRFSSRKVSPKVPTG     240
Bd3-1     SKNISRSSSGYIGKLRSNFLGTKFIIYDTQPPYNAGKLCSQERASRRFSSRKVSPKVPTG     240
Bd21      SKNISRSSSGYIGKLRSNFLGTKFIIYDTQPPYNAGKLCSQERASRRFSSRKVSPKVPTG     240
Bd30-1    SKNISRSSSGYIGKLRSNFLGTKFIIYDTQPPYNAGKLCSQERASRRFSSRKVSPKVPTG     240
Tek-4     SKNISRSSSGYIGKLRANFLGTKFIIYDTQPPYNAGKLCSQERASRRFSSRKVSPKVPTG     240
ABR6      SKNISRSSSGYIGKLRSNFLGTKFIIYDTQPPYNAGKLCSQERASRRFSSRKVSPKVPTG     240
Bd29-1    SKNISRSSSGYIGKLRSNFLGTKFIIYDTQPPYNAGKLCSQERASRRFSSRKVSPKVPTG     240
Bd1-1     SKNISRSSSGYIGKLRSNFLGTKFIIYDTQPPYNAGKLCSQERASRRFSSRKVSPKVPTG     240
RON2      SKNISRSSSGYIGKLRSNFLGTKFIIYDTQPPYNAGKLCSQERASRRFSSRKVSPKVPTG     240
                          :**** **************************************
```

```
Koz-3       SYPIAQVNYELNVLGTRGPRRMQCTMHSIPRSAVDPDGTVPGQPKELLPRLFDESFRTTT    275
Bd21-3      SYPIAQVNYELNVLGTRGPRRMQCTMHSIPRSAVDPDGTVPGQPKELLPRLFDESFRTTT    275
Bd2-3       SYPIAQVNYELNVLGTRGPRRMQCTMHSIPRSAVDPDGTVPGQPKELLPRLFDESFRTTT    300
Bd3-1       SYPIAQVNYELNVLGTRGPRRMQCTMHSIPRSAVDPDGTVPGQPKELLPRLFDESFRTTT    300
Bd21        SYPIAQVNYELNVLGTRGPRRMQCTMHSIPRSAVDPDGTVPGQPKELLPRLFDESFRTTT    300
Bd30-1      SYPIAQVNYELNVLGTRGPRRMQCTMHSIPRSAVDPDGTVPGQPKELLPRLFDESFRTTT    300
Tek-4       SYPIAQVNYELNVLGTRGPRRMQCTMHSIPRSAVDPDGTVPGQPKELLPRLFDESFRTTT    300
ABR6        SYPIAQVNYELNVLGTRGPRRMQCTMHSIPRSAVDPDGTVPGQPKELLPRLFDESFRTTT    300
Bd29-1      SYPIAQVNYELNVLGTRGPRRMQCTMHSIPRSAVDPDGTVPGQPKELLPRLFDESFRTTT    300
Bd1-1       SYPIAQVNYELNVLGTRGPRRMQCTMHSIPRSAVDPDGTVPGQPKELLPRLFDESFRTTT    300
RON2        SYPIAQVNYELNVLGTRGPRRMQCTMHSIPRSAVDPDGTVPGQPKELLPRLFDESFRTTT    300
            ************************************************************

Koz-3       SSFSKYSVADHSTDFGSSRFSEYGGGALQDGDAADKETPLVLRNKVPRWHEQLQCWCLNF    335
Bd21-3      SSFSKYSVADHSTDFGSSRFSEYGGGALQDGDAADKETPLVLRNKVPRWHEQLQCWCLNF    335
Bd2-3       SSFSKYSVADHSTDFGSSRFSEYGGGALQDGDAADKETPLVLRNKVPRWHEQLQCWCLNF    360
Bd3-1       SSFSKYSVADHSTDFGSSRFSEYGGGALQDGDAADKETPLVLRNKVPRWHEQLQCWCLNF    360
Bd21        SSFSKYSVADHSTDFGSSRFSEYGGGALQDGDAADKETPLVLRNKVPRWHEQLQCWCLNF    360
Bd30-1      SSFSKYSVADHSTDFGSSRFSEFGGGALQDGDAADKETPLVLRNKAPRWHEQLQCWCLNF    360
Tek-4       SSFSKYSVADHSTDFGSSRFSEFGGGALQDGDAADKETPLVLRNKAPRWHEQLQCWCLNF    360
ABR6        SSFSKYSVADHSTDFGSSRFSEFGGGALQDGDAADKETPLVLRNKAPRWHEQLQCWCLNF    360
Bd29-1      SSFSKYSVADHSTDFGSSRFSEFGGGALQDGDAADKETPLVLRNKAPRWHEQLQCWCLNF    360
Bd1-1       SSFSKYSVADHSTDFGSSRFSEFGGGALQDGDAADKETPLVLRNKAPRWHEQLQCWCLNF    360
RON2        SSFSKYSVADHSTDFGSSRFSEFGGGALQDGDAADKETPLVLRNKAPRWHEQLQCWCLNF    360
            **********************:**********************.**************

Koz-3       RGRVTVASVKNFQLIAGAPLPAPVASSEPSQPAPAQQPAQPQPSSSSSSSSHDTVLLQFG    395
Bd21-3      RGRVTVASVKNFQLIAGAPLPAPVASSEPSQPAPAQQPAQPQPSSSSSSSSHDTVLLQFG    395
Bd2-3       RGRVTVASVKNFQLIAGAPLPAPVASSEPSQPAPAQQPAQPQPSSSSSSSSHDTVLLQFG    420
Bd3-1       RGRVTVASVKNFQLIAGAPLPAPVASSEPPQPAPAQQPAQPQPSSSSSSSSHDTVLLQFG    420
Bd21        RGRVTVASVKNFQLIAGAPLPAPVASSEPSQPAPAQQPAQPQPSSSSSSSSHDTVLLQFG    420
Bd30-1      RGRVTVASVKNFQLIAGAPPPAPVASSEPPQPAPAQQPAQPQPSSSSSSPSHDTVLLQFG    420
Tek-4       RGRVTVASVKNFQLIAGAPPPAPVASSEPSQPAPAQQPAQPQPSSSSSSSSHDTVLLQFG    420
ABR6        RGRVTVASVKNFQLIAGAPPPAPVASSEPSQPAPAQQPAQPQPSSSSSSSSHDTVLLQFG    420
Bd29-1      RGRVTVASVKNFQLIAGAPPPAPVASSEPSQPAPAQQPAQPQPSSSSSSSSHDTVLLQFG    420
Bd1-1       RGRVTVASVKNFQLIAGAPPPAPVASSEPSQPAPAQQPAQPQPSSSSSSSSHDTVLLQFG    420
RON2        RGRVTVASVKNFQLIAGAPPPAPVASSEPSQPAPAQQPAQPQPSSSSSSSSHDTVLLQFG    420
            ******************* ********* ******************* **********

Koz-3       KVSKDTFTMDYRYPLSAFQAFAICLTSFDTKLACE 430
Bd21-3      KVSKDTFTMDYRYPLSAFPALAICLTSFDTKLACE 430
Bd2-3       KVSKDTFTMDYRYPLSAFQAFAICLTSFDTKLACE 455
Bd3-1       KVSKDTFTMDYRYPLSAFQAFAICLTSFDTKLACE 455
Bd21        KVSKDTFTMDYRYPLSAFQAFAICLTSFDTKLACE 455
Bd30-1      KVSKDTFTMDYRYPLSAFQAFAICLTSFDTKLACE 455
Tek-4       KVSKDTFTMDYRYPLSAFQAFAICLTSFDTKLACE 455
ABR6        KVSKDTFTMDYRYPLSAFQAFAICLTSFDTKLACE 455
Bd29-1      KVSKDTFTMDYRYPLSAFQAFAICLTSFDTKLACE 455
Bd1-1       KVSKDTFTMDYRYPLSAFQAFAICLTSFDTKLACE 455
RON2        KVSKDTFTMDYRYPLSAFQAFAICLTSFDTKLACE 455
            ****************** *:**************
```

BdTLP4

```
Bd21-3      MSFRSIVRDVRDSFGSLSRRGFEVRFLGHRRGKSHSAVHELHDPAPVIQSSCWANLPPEL      60
Tek-4       MSFRSIVRDVRDSFGSLSRRGFEVRFLGHRRGKSHSAVHELHDPAPVIQSSCWANLPPEL      60
Bd29-1      MSFRSIVRDVRDSFGSLSRRGFEVRFLGHRRGKSHSAVHELHDPAPVIQSSCWANLPPEL      60
Bd1-1       MSFRSIVRDVRDSFGSLSRRGFEVRFLGHRRGKSHSAVHELHDPAPVIQSSCWANLPPEL      60
Bd3-1       MSFRSIVRDVRDSFGSLSRRGFEVRFLGHRRGKSHSAVHELHDPAPVIQSSCWANLPPEL      60
Bd21        MSFRSIVRDVRDSFGSLSRRGFEVRFLGHRRGKSHSAVHELHDPAPVIQSSCWANLPPEL      60
ABR6        MSFRSIVRDVRDSFGSLSRRGFEVRFLGHRRGKSHSAVHELHDPAPVIQSSCWANLPPEL      60
Bd30-1      MSFRSIVRDVRDSFGSLSRRGFEVRFLGHRRGKSHSAVHELHDPAPVIQSSCWANLPPEL      60
Bd2-3       MSFRSIVRDVRDSFGSLSRRGFEVRFLGHRRGKSHSAVHELHDPAPVIQSSCWANLPPEL      60
RON2        MSFRSIVRDVRDSFGSLSRRGFEVRFLGHRRGKSHSAVHELHDPAPVIQSSCWANLPPEL      60
Koz-3       MSFRSIVRDVRDSFGSLSRRGFEVRFLGHRRGKSHSAVHELHDPAPVIQSSCWANLPPEL      60
            ************************************************************

Bd21-3      LRDVIERLEASEATWPSRKNVVACAAVCRTWREMCKEIVKNPEFCGKITFPVSLKQPGPR      120
Tek-4       LRDVIERLEASEATWPSRKNVVACAAVCRTWREMCKEIVKNPEFCGKITFPVSLKQPGPR      120
Bd29-1      LRDVIERLEASEATWPSRKNVVACAAVCRTWREMCKEIVKNPEFCGKITFPVSLKQPGPR      120
Bd1-1       LRDVIERLEASEATWPSRKNVVACAAVCRTWREMCKEIVKNPEFCGKITFPVSLKQPGPR      120
Bd3-1       LRDVIERLEASEATWPSRKNVVACAAVCRTWREMCKEIVKNPEFCGKITFPVSLKQPGPR      120
Bd21        LRDVIERLEASEATWPSRKNVVACAAVCRTWREMCKEIVKNPEFCGKITFPVSLKQPGPR      120
ABR6        LRDVIERLEASEATWPSRKNVVACAAVCRTWREMCKEIVKNPEFCGKITFPVSLKQPGPR      120
Bd30-1      LRDVIERLEASEATWPSRKNVVACAAVCRTWREMCKEIVKNPEFCGKITFPVSLKQPGPR      120
Bd2-3       LRDVIERLEASEATWPSRKNVVACAAVCRTWREMCKEIVKNPEFCGKITFPVSLKQPGPR      120
RON2        LRDVIERLEASEATWPSRKNVVACAAVCRTWREMCKEIVKNPEFCGKITFPVSLKQPGPR      120
Koz-3       LRDVIERLEASEATWPSRKNVVACAAVCRTWREMCKEIVKNPEFCGKITFPVSLKQPGPR      120
            ************************************************************

Bd21-3      DGTIQCFIKRDKSTQTYYLYLCLSSAVLVESGKFLLLAKRYSRPTCTEYTIFMNADNTSR      180
Tek-4       DGTIQCFIKRDKSTQTYYLYLCLSSAVLVESGKFLLLAKRYSRPTCTEYTIFMNADNTSR      180
Bd29-1      DGTIQCFIKRDKSTQTYYLYLCLSSAVLVESGKFLLLAKRYSRPTCTEYTIFMNADNTSR      180
Bd1-1       DGTIQCFIKRDKSTQTYYLYLCLSSAVLVESGKFLLLAKRSSRPTCTEYTIFMNADNTSR      180
Bd3-1       DGTIQCFIKRDKSTQTYYLYLCLSSAVLVESGKFLLLAKRYSRPTCTEYTIFMNADNTSR      180
Bd21        DGTIQCFIKRDKSTQTYYLYLCLSSAVLVESGKFLLLAKRYSRPTCTEYTIFMNADNTSR      180
ABR6        DGTIQCFIKRDKSTQTYYLYLCLSSAVLVESGKFLLLAKRYSRPTCTEYTIFMNADNTSR      180
Bd30-1      DGTIQCFIKRDKSTQTYYLYLCLSSAVLVESGKFLLLAKRYSRPTCTEYTIFMNADNTSR      180
Bd2-3       DGTIQCFIKRDKSTQTYYLYLCLSSAVLVESGKFLLLAKRYSRPTCTEYTIFMNADNTSR      180
RON2        DGTIQCFIKRDKSTQTYYLYLCLSSAVLVESGKFLLLAKRYSRPTCTEYTIFMNADNTSR      180
Koz-3       DGTIQCFIKRDKSTQTYYLYLCLSSAVLVESGKFLLLAKRYSRPTCTEYTIFMNADNTSR      180
            **************************************** *******************

Bd21-3      SSNMYIGKLRSNLLGTKFVIYDPQPPCNIPSVSQPGKTSRRFYSRKVSLKAPSSTYSIAQ      240
Tek-4       SSNMYIGKLRSNLLGTKFVIYDTQPPCNIPSVSQPGKTSRRFYSRKVSLKAPSSTYSIAQ      240
Bd29-1      SSNMYIGKLRSNLLGTKFVIYDTQPPCNIPSVSQPGKTSRRFYSRKVSLKAPSSTYSIAQ      240
Bd1-1       SSNMYIGKLRSNLLGTKFVIYDTQPPCNIPSVSQPGKTSRRFYSRKVSLKAPSSTYSIAQ      240
Bd3-1       SSNMYIGKLRSNLLGTKFVIYDTQPPCNIPSVSQPGKTSRRFYSRKVSLKAPSSTYSIAQ      240
Bd21        SSNMYIGKLRSNLLGTKFVIYDTQPPCNIPSVSQPGKTSRRFYSRKVSLKAPSSTYSIAQ      240
ABR6        SSNMYIGKLRSNLLGTKFVIYDTQPPCNIPSVSQPGKTSRRFYSRKVSLKAPSSTYSIAQ      240
Bd30-1      SSNMYIGKLRSNLLGTKFVIYDTQPPCNIPSVSQPGKTSRRFYSRKVSLKAPSSTYSIAQ      240
Bd2-3       SSNMYIGKLRSNLLGTKFVIYDTQPPCNIPSVSQPGKTSRRFYSRKVSLKAPSSTYSIAQ      240
RON2        SSNMYIGKLRSNLLGTKFVIYDTQPPCNIPSVSQPGKTSRRFYSRKVSLKAPSSTYSIAQ      240
Koz-3       SSNMYIGKLRSNLLGTKFVIYDTQPPCNIPSVSQPGKTSRRFYSRKVSLKAPSSTYSIAQ      240
            ********************** *************************************
```

```
Bd21-3      VSYELNVLGTRGPRRMNCVMHPIPASSLEAGGTVPCQPDSVLARSIDESFGSISFSKSSI      300
Tek-4       VSYELNVLGTRGPRRMNCVMHSIPASSLEAGGTVPCQPDSVLARSIDESFGSISFSKSSI      300
Bd29-1      VSYELNVLGTRGPRRMNCVMHSIPASSLEAGGTVPCQPDSVLARSIDESFGSISFSKSSI      300
Bd1-1       VSYELNVLGTRGPRRMNCVMHSIPASSLEAGGTVPCQPDSVLARSIDESFGSISFSKSSI      300
Bd3-1       VSYELNVLGTRGPRRMNCVMHSIPASSLEAGGTVPCQPDSVLARSIDESFGSISFSKSSI      300
Bd21        VSYELNVLGTRGPRRMNCVMHSIPASSLEAGGTVPCQPDSVLARSIDESFGSISFSKSSI      300
ABR6        VSYELNVLGTRGPRRMNCVMHSIPASSLEAGGTVPCQPDSVLARSIDESFGSISFSKSSI      300
Bd30-1      VSYELNVLGTRGPRRMNCVMHSIPASSLEAGGTVPCQPDSVLARSIDESFGSISFSKSSI      300
Bd2-3       VSYELNVLGTRGPRRMNCVMHSIPASSLEAGGTVPCQPDSVLARSIDESFGSISFSKSSI      300
RON2        VSYELNVLGTRGPRRMNCVMHSIPASSLEAGGTVPCQPDSVLARSIDESFGSISFSKSSI      300
Koz-3       VSYELNVLGTRGPRRMNCVMHSIPASSLEAGGTVPCQPDSVLARSIDESFGSISFSKSSI      300
            ********************* **************************************

Bd21-3      RDRSIRFSSTRFSDISVGCSRNGGQTLGDNNESKECPLILHNKAPRWHEQLQCWCLNFKG      360
Tek-4       RDRSIRFSSTRFSDISVGCSRNGGQTLGDNNESKECPLILHNKAPRWHEQLQCWCLNFKG      360
Bd29-1      RDRSIRFSSTRFSDISVGCSRNGGQTLGDNNESKECPLILHNKAPRWHEQLQCWCLNFKG      360
Bd1-1       RDRSIRFSSTRFSDISVGCSRNGGQTLGDNNESKECPLILRNKAPRWHEQLQCWCLNFKG      360
Bd3-1       RDRSIRFSSTRFSDISVGCSRNGGQTLGDNNESKECPLILRNKAPRWHEQLQCWCLNFKG      360
Bd21        RDRSIRFSSTRFSDISVGCSRNGGQTLGDNNESKECPLILRNKAPRWHEQLQCWCLNFKG      360
ABR6        RDRSIRFSSTRFSDISVGCSRNGGQTLGDNNESKECPLILRNKAPRWHEQLQCWCLNFKG      360
Bd30-1      RDRSIRFSSTRFSDISVGCSRNGGQTLGDNNESKECPLILRNKAPRWHEQLQCWCLNFKG      360
Bd2-3       RDRSIRFSSTRFSDISVGCSRNGGQTLGDNNESKECPLILRNKAPRWHEQLQCWCLNFKG      360
RON2        RDRSIRFSSTRFSDISVGCSRNGGQTLGDNNESKECPLILRNKAPRWHEQLQCWCLNFKG      360
Koz-3       RDRSIRFSSTRFSDISVGCSRNGGQTLGDNNESKECPLILRNKAPRWHEQLQCWCLNFKG      360
            ****************************************:*******************

Bd21-3      RVTVASVKNFQLVASTQPAAAAPAPSPPAPTPPSEHDKVILQFGKVAKDMFTMDYRYPLS      420
Tek-4       RVTVASVKNFQLVASTQPAAAAPAPSQPAPTPPSEHDKVILQFGKVAKDMFTMDYRYPLS      420
Bd29-1      RVTVASVKNFQLVASTQPAAAAPAPSQPAPTPPSEHDKVILQFGKVAKDMFTMDYRYPLS      420
Bd1-1       RVTVASVKNFQLVASTQPAAAAPAPSQPAPTLPSEHDKVILQFGKVAKDMFTMDYRYPLS      420
Bd3-1       RVTVASVKNFQLVASTQPAAAAPAPSPPAPTLPSEHDKVILQFGKVAKDMFTMDYRYPLS      420
Bd21        RVTVASVKNFQLVASTQPAAAAPAPSQPAPTLPSEHDKVILQFGKVAKDMFTMDYRYPLS      420
ABR6        RVTVASVKNFQLVASTQPAAAAPAPSQPAPTLPSEHDKVILQFGKVAKDMFTMDYRYPLS      420
Bd30-1      RVTVASVKNFQLVASTQPAAAAPAPSQPAPTLPSEHDKVILQFGKVAKDMFTMDYRYPLS      420
Bd2-3       RVTVASVKNFQLVASTQPAAAAPAPSQPAPTLPSEHDKVILQFGKVAKDMFTMDYRYPLS      420
RON2        RVTVASVKNFQLVASTQPAAAAPAPSQPAPTLPSEHDKVILQFGKVAKDMFTMDYRYPLS      420
Koz-3       RVTVASVKNFQLVASTQPAAAAPAPSQPAPTLPSEHDKVILQFGKVAKDMFTMDYRYPLS      420
            ************************** **** ****************************

Bd21-3      AFQAFAICLSSFDTKLACE 439
Tek-4       AFQAFAICLSSFDTKLACE 439
Bd29-1      AFQAFAICLSSFDTKLACE 439
Bd1-1       AFQAFAICLSSFDTKLACE 439
Bd3-1       AFQAFAICLSSFDTKLACE 439
Bd21        AFQAFAICLSSFDTKLACE 439
ABR6        AFQAFAICLSSFDTKLACE 439
Bd30-1      AFQAFAICLSSFDTKLACE 439
Bd2-3       AFQAFAICLSSFDTKLACE 439
RON2        AFQAFAICLSSFDTKLACE 439
Koz-3       AFQAFAICLSSFDTKLACE 439
            *******************
```

BdTLP5

```
Bd3-1       MSFRSIARDVRDGFGSLSRRSFEVTIASLYGLTGYHKGKAQSSLDDLHDPPPIIQESRWA    60
Koz-3       MSFRSIARDVRDGFGSLSRRSFEVTIASLYGLTGYHKGKAQSSLDDLHDPPPIIQESRWA    60
Bd21        MSFRSIARDVRDGFGSLSRRSFEVTIASLYGLTGYHKGKAQSSLDDLHDPPPIIQESRWA    60
Tek-4       MSFRSIARDVRDGFGSLSRRSFEVTIASLYGLTGYHKGKAQSSLDDLHDPPPIIQESRWA    60
ABR6        MSFRSIARDVRDGFGSLSRRSFEVTIASLYGLTGYHKGKAQSSLDDLHDPPPIIQESRWA    60
Bd30-1      MSFRSIARDVRDGFGSLSRRSFEVTIASLYGLTGYHKGKAQSSLDDLHDPPPIIQESRWA    60
Bd29-1      MSFRSIARDVRDGFGSLSRRSFEVTIASLYGLTGYHKGKAQSSLDDLHDPPPIIQESRWA    60
Bd1-1       MSFRSIARDVRDGFGSLSRRSFEVTIASLYGLTGYHKGKAQSSLDDLHDPPPIIQESRWA    60
Bd2-3       MSFRSIARDVRDGFGSLSRRSFEVTIASLYGLTGYHKGKAQSSLDDLHDPPPIIQESRWA    60
RON2        MSFRSIARDVRDGFGSLSRRSFEVTIASLYGLTGYHKGKAQSSLDDLHDPPPIIQESRWA    60
Bd21-3      MSFRSIARDVRDGFGSLSRRSFEVTIASLYGLTGYHKGKAQSSLDDLHDPPPIIQESRWA    60
            ************************************************************

Bd3-1       SLPPELLRDVIRRLESDESTWPSRKHVVCFAAVCKTWREMCREIVLSPEFCGKLTFRVSL    120
Koz-3       SLPPELLRDVIRRLESDESTWPSRKHVVCFAAVCKTWREMCREIVLSPEFCGKLTFRVSL    120
Bd21        SLPPELLRDVIRRLESDESTWPSRKHVVCFAAVCKTWREMCREIVLSPEFCGKLTFRVSL    120
Tek-4       SLPPELLRDVIRRLESDESTWPSRKHVVCFAAVCKTWREMCREIVLSPEFCGKLTFRVSL    120
ABR6        SLPPELLRDVIRRLESDESTWPSRKHVVCFAAVCKTWREMCREIVLSPEFCGKLTFRVSL    120
Bd30-1      SLPPELLRDVIRRLESDESTWPSRKHVVCFAAVCKTWREMCREIVLSPEFCGKLTFRVSL    120
Bd29-1      SLPPELLRDVIRRLESDESTWPSRKHVVCFAAVCKTWREMCREIVLSPEFCGKLTFRVSL    120
Bd1-1       SLPPELLRDVIRRLESDESTWPSRKHVVCFAAVCKTWREMCREIVLSPEFCGKLTFRVSL    120
Bd2-3       SLPPELLRDVIRRLESDESTWPSRKHVVCFAAVCKTWREMCREIVLSPEFCGKLTFRVSL    120
RON2        SLPPELLRDVIRRLESDESTWPSRKHVVCFAAVCKTWREMCREIVLSPEFCGKLTFRVSL    120
Bd21-3      SLPPELLRDVIRRLESDESTWPSRKHVVCFAAVCKTWREMCREIVLSPEFCGKLTFRVSL    120
            ************************************************************

Bd3-1       KQPGPREGMIQCFIKRNKSKSTYQLYLCLSNVVTAETGKFLLSAKRNRKTSCTEYAISMD    180
Koz-3       KQPGPREGMIQCFIKRNKSKSTYQLYLCLSNVVTAETGKFLLSAKRNRKTSCTEYAISMD    180
Bd21        KQPGPREGMIQCFIKRNKSKSTYQLYLCLSNVVTAETGKFLLSAKRNRKTSCTEYAISMD    180
Tek-4       KQPGPREGMIQCFIKRNKSKSTYQLYLCLSNVVTAETGKFLLSAKRNRKTSCTEYAISMD    180
ABR6        KQPGPREGMIQCFIKRNKSKSTYQLYLCLSNVVTAETGKFLLSAKRNRKTSCTEYAISMD    180
Bd30-1      KQPGPREGMIQCFIKRNKSKSTYQLYLCLSNVVTAETGKFLLSAKRNRKTSCTEYAISMD    180
Bd29-1      KQPGPREGMIQCFIKRNKSKSTYQLYLCLSNVVTAETGKFLLSAKRNRKTSCTEYAISMD    180
Bd1-1       KQPGPREGMIQCFIKRNKSKSTYQLYLCLSNVVTAETGKFLLSAKRNRKTSCTEYAISMD    180
Bd2-3       KQPGPREGMIQCFIKRNKSKSTYQLYLCLSNVVTAETGKFLLSAKRNRKTSCTEYAISMD    180
RON2        KQPGPREGMIQCFIKRNKSKSTYQLYLCLSNVVTAETGKFLLSAKRNRKTSCTEYAISMD    180
Bd21-3      KQPGPREGMIQCFIKRNKSKSTYQLYLCLSNVVTAETGKFLLSAKRNRKTSCTEYAISMD    180
            ************************************************************

Bd3-1       TGNTSRSNRTYIGKLRSNFLGTKFLIYDTQPPYNGAVVPPVGRTSRRFNSTKVSPKVPAG    240
Koz-3       TGNTSRSNRTYIGKLRSNFLGTKFLIYDTQPPYNGAVVPPVGRTSRRFNSTKVSPKVPAG    240
Bd21        TGNTSRSNRTYIGKLRSNFLGTKFLIYDTQPPYNGAVVPPVGRTSRRFNSTKVSPKVPAG    240
Tek-4       TGNTSRSNRTYIGKLRSNFLGTKFLIYDTQPPYNGAVVPPVGRTSRRFNSTKVSPKVPAG    240
ABR6        TGNTSRSNRTYIGKLRSNFLGTKFLIYDTQPPYNGAVVPPVGRTSRRFNSTKVSPKVPAG    240
Bd30-1      TGNTSRSNRTYIGKLRSNFLGTKFLIYDTQPPYNGAVVPPVGRTSRRFNSTKVSPKVPAG    240
Bd29-1      TGNTSRSNRTYIGKLRSNFLGTKFLIYDTQPPYNGAVVPPVGRTSRRFNSTKVSPKVPAG    240
Bd1-1       TGNTSRSNRTYIGKLRSNFLGTKFLIYDTQPPYNGAVVPPVGRTSRRFNSTKVSPKVPAG    240
Bd2-3       TGNTSRSNRTYIGKLRSNFLGTKFLIYDTQPPYNGAVVPPVGRTSRRFNSTKVSPKVPAG    240
RON2        TGNTSRSNRTYIGKLRSNFLGTKFLIYDTQPPYNGAVVPPVGRTSRRFNSTKVSPKVPAG    240
Bd21-3      TGNTSRSNRTYIGKLRSNFLGTKFLIYDTQPPYNGAVVPPVGRTSRRFNSTKVSPKVPAG    240
            ************************************************************
```

```
Bd3-1      SYNIAQVTYELNVLGTRGPRRMRCIMHSIPASSVEPGGIVPGQPEQIVPRALGDSFRSIS      300
Koz-3      SYNIAQVTYELNVLGTRGPRRMRCIMHSIPASSVEPGGIVPGQPEQIVPRALGDSFRSIS      300
Bd21       SYNIAQVTYELNVLGTRGPRRMRCIMHSIPASSVEPGGIVPGQPEQIVPRALGDSFRSIS      300
Tek-4      SYNIAQVTYELNVLGTRGPRRMRCIMHSIPASSVEPGGIVPGQPEQIVPRALGDSFRSIS      300
ABR6       SYNIAQVTYELNVLGTRGPRRMRCIMHSIPASSVEPGGIVPGQPEQIVPRALGDSFRSIS      300
Bd30-1     SYNIAQVTYELNVLGTRGPRRMRCIMHSIPASSVEPGGIVPGQPEQIVPRALGDSFRSIS      300
Bd29-1     SYNIAQVTYELNVLGTRGPRRMRCIMHSIPASSVEPGGIVPGQPEQIVPRALGDSFRSIS      300
Bd1-1      SYNIAQVTYELNVLGTRGPRRMRCIMHSIPASSVEPGGIVPGQPEQIVPRALGDSFRSIS      300
Bd2-3      SYNIAQVTYELNVLGTRGPRRMRCIMHSIPASSVEPGGIVPGQPEQIVPRALGDSFRSIS      300
RON2       SYNIAQVTYELNVLGTRGPRRMRCIMHSIPASSVEPGGIVPGQPEQIVPRALGDSFRSIS      300
Bd21-3     SYNIAQVTYELNVLGTRGPRRMRCIMHSIPASSVEPGGIVPGQPEQIVPRALGDSFRSIS      300
           ************************************************************

Bd3-1      SLSQSFRSSTTFSKSIMDQSMDFNSARFSEINGGIVDGDGDCEVKDRPLVLRNKPPRWHE      360
Koz-3      SLSQSFRSSTTFSKSIMDQSMDFNSARFSEINGGIVDGDGDCEVKDRPLVLRNKPPRWHE      360
Bd21       SLSQSFRSSTTFSKSIMDQSMDFNSARFSEINGGIVDGDGDCEVKDRPLVLRNKPPRWHE      360
Tek-4      SLSQSFRSSTTFSKSIMDQSMDFNSARFSEINGGIVDGDGDCEVKDRPLVLRNKPPRWHE      360
ABR6       SLSQSFRSSTTFSKSIMDQSMDFNSARFSEINGGIVDGDGDCEVKDRPLVLRNKPPRWHE      360
Bd30-1     SLSQSFRSSTTFSKSIMDQSMDFNSARFSEINGGIVDGDGDCEVKDRPLVLRNKPPRWHE      360
Bd29-1     SLSQSFRSSTTFSKSIMDQSMDFNSARFSEINGGIVDGDGDCEVKDRPLVLRNKPPRWHE      360
Bd1-1      SLSQSFRSSTTFSKSIMDQSMDFNSARFSEINGGIVDGDGDCEVKDRPLVLRNKPPRWHE      360
Bd2-3      SLSQSFRSSTTFSKSIMDQSMDFNSARFSEINGGIVDGDGDCEVKDRPLVLRNKPPRWHE      360
RON2       SLSQSFRSSTTFSKSIMDQSMDFNSARFSEINGGIVDGDGDCEVKDRPLVLRNKPPRWHE      360
Bd21-3     SLSQSFRSSTTFSKSIMDQSMDFNSARFSEINGGIVEGDGDCEVKDRPLVLRNKPPRWHE      360
           ************************************:***********************

Bd3-1      QLQCWCLNFRGRVTIASVKNFQLIAASNPPPAGAPTPSQPGPVDPDKVILQFGKVARDMF      420
Koz-3      QLQCWCLNFRGRVTIASVKNFQLIAASNPPPAGAPTPSQPGPVDPDKVILQFGKVARDMF      420
Bd21       QLQCWCLNFRGRVTIASVKNFQLIAASNPPPAGAPTPSQPGPVDPDKVILQFGKVARDMF      420
Tek-4      QLQCWCLNFRGRVTIASVKNFQLIAASNPPPAGAPTPSQPGPVDPDKVILQFGKVARDMF      420
ABR6       QLQCWCLNFRGRVTIASVKNFQLIAASNPPPAGAPTPSQPGPVDPDKVILQFGKVARDMF      420
Bd30-1     QLQCWCLNFRGRVTIASVKNFQLIAASNPPPAGAPTPSQPGPVDPDKVILQFGKVARDMF      420
Bd29-1     QLQCWCLNFRGRVTIASVKNFQLIAASNPPPAGAPTPSQPGPVDPDKVILQFGKVARDMF      420
Bd1-1      QLQCWCLNFRGRVTIASVKNFQLIAASNPPPAGAPTPSQPGPVDPDKVILQFGKVARDMF      420
Bd2-3      QLQCWCLNFRGRVTIASVKNFQLIAASNPPPAGAPTPSQPGPVDPDKVILQFGKVARDMF      420
RON2       QLQCWCLNFRGRVTIASVKNFQLIAASNPPPAGAPTPSQPGPVDPDKVILQFGKVARDMF      420
Bd21-3     QLQCWCLNFRGRVTIASVKNFQLIAASNPPPAGAPTPSQPGPVDPDKVILQFGKVARDMF      420
           ************************************************************

Bd3-1      TMDYRYPLSAFQAFAICLSSFDTKLACE        448
Koz-3      TMDYRYPLSAFQAFAICLSSFDTKLACE        448
Bd21       TMDYRYPLSAFQAFAICLSSFDTKLACE        448
Tek-4      TMDYRYPLSAFQAFAICLSSFDTKLACE        448
ABR6       TMDYRYPLSAFQAFAICLSSFDTKLACE        448
Bd30-1     TMDYRYPLSAFQAFAICLSSFDTKLACE        448
Bd29-1     TMDYRYPLSAFQAFAICLSSFDTKLACE        448
Bd1-1      TMDYRYPLSAFQAFAICLSSFDTKLACE        448
Bd2-3      TMDYRYPLSAFQAFAICLSSFDTKLACE        448
RON2       TMDYRYPLSAFQAFAICLSSFDTKLACE        448
Bd21-3     TMDYRYPLSAFQAFAICLSSFDTKLACE        448
           ****************************
```

BdTLP6

```
Bd21        MSFRSIVRDVKDGFGSLSQRRFETRLLGHCRGKSHGAVHELHDPVPVIQSSCWANLPPEL    60
Bd30-1      MSFRSIVRDVKDGFGSLSQRRFETRLLGHCRGKSHGAVHELHDPVPVIQSSCWANLPPEL    60
RON2        MSFRSIVRDVKDGFGSLSQRRFETRLLGHCRGKSHGAVHELHDPVPVIQSSCWANLPPEL    60
Bd21-3      MSFRSIVRDVKDGFGSLSQRRFETRLLGHCRGKSHGAVHELHDPVPVIQSSCWANLPPEL    60
ABR6        MSFRSIVRDVKDGFGSLSQRRFETRLLGHCRGKSHGAVHELHDPVPVIQSSCWANLPPEL    60
Bd3-1       MSFRSIVRDVKDGFGSLSQRRFETRLLGHCRGKSHGAVHELHDPVPVIQSSCWANLPPEL    60
Bd1-1       MSFRSIVRDVKDGFGSLSQRRFETRLLGHCRGKSHGAVHELHDPVPVIQSSCWANLPPEL    60
Bd2-3       MSFRSIVRDVKDGFGSLSQRRFETRLLGHCRGKSHGAVHELHDPVPVIQSSCWANLPPEL    60
Bd29-1      MSFRSIVRDVKDGFGSLSQRRFETRLLGHCRGKSHGAVHELHDPVPVIQSSCWANLPPEL    60
Tek-4       MSFRSIVRDVKDGFGSLSQRRFETRLLGHCRGKSHGAVHELHDPVPVIQSSCWANLPPEL    60
Koz-3       MSFRSIVRDVKDGFGSLSQRRFETRLLGHCRGKSHGAVHELHDPVPVIQSSCWANLPPEL    60
            ************************************************************

Bd21        LRDVIERLEASEATWPCRRNVVACAAVCKTWREMCKEIVKNPEECGKLTFPVSLKQPGPR    120
Bd30-1      LRDVIERLEASEATWPCRRNVVACAAVCKTWREMCKEIVKNPEECGKLTFPVSLKQPGPR    120
RON2        LRDVIERLEASEATWPCRRNVVACAAVCKTWREMCKEIVKNPEECGKLTFPVSLKQPGPR    120
Bd21-3      LRDVIERLEASEATWPCRRNVVACAAVCKTWREMCKEIVKNPEECGKLTFPVSLKQPGPR    120
ABR6        LRDVIERLEASEATWPCRRNVVACAAVCKTWREMCKEIVKNPEECGKLTFPVSLKQTGPR    120
Bd3-1       LRDVIERLEASEATWPCRRNVVACAAVCKTWREMCKEIVKNPEECGKLTFPVSLKQPGPR    120
Bd1-1       LRDVIERLEASEATWPCRRNVVACAAVCKTWREMCKEIVKNPEECGKLTFPVSLKQPGPR    120
Bd2-3       LRDVIERLEASEATWPCRRNVVACAAVCKTWREMCKEIVKNPEECGKLTFPVSLKQPGPR    120
Bd29-1      LRDVIERLEASEATWPCRRNVVACAAVCKTWREMCKEIVKNPEECGMLTFPVSLKQPGPR    120
Tek-4       LRDVIERLEASEATWPCRRNVVACAAVCKTWREMCKEIVKNPEECGKLTFPVSLKQPGPR    120
Koz-3       LRDVIERLEASEATWPCRRNVVACAAVCKTWREMCKEIVKNPEECGKLTFPVSLKQPGPR    120
            *********************************************** ******** ***

Bd21        HGIIQCFIKRDRSTQTYYLYLSLSSAVLVENGKFLLSAKRICRATWTEYIISMNANSISS    180
Bd30-1      DGIIQCFIKRDRSTQTYYLYLSLSSAVLVENGKFLLSAKRICRATWTEYIISMNANSISS    180
RON2        DGIIQCFIKRDRSTQTYYLYLSLSSAVLVENGKFLLSAKRICRATWTEYIISMNANSISS    180
Bd21-3      DGIIQCFIKRDRSTQTYYLYLSLSSAVLVENGKFLLSAKRICRATWTEYIISMNANSISS    180
ABR6        DGIIQCFIKRDRSTQTYYLYLSLSSAVLVENGKFLLSAKRICRATWTEYIISMNANSISS    180
Bd3-1       DGIIQCFIKRDRSTQTYYLYLSLSSAVLVENGKFLLSAKRICRATWTEYIISMNANSISS    180
Bd1-1       DGIIQCFIKRDRSTQTYYLYLSLSSAVLVENGKFLLSAKRICRATWTEYIISMNANSISS    180
Bd2-3       DGIIQCFIKRDRSTQTYYLYLSLSSAVLVENGKFLLSAKRICRATWTEYIISMNANSISS    180
Bd29-1      DGIIQCFIKRDRSTQTYYLYLSLSSAVLVENGKFLLSAKRICRATWTEYIISMNANSISS    180
Tek-4       DGIIQCFIKRDRSTQTYYLYLSLSSAVLVENGKFLLSAKRICRATWTEYIISMNANSISS    180
Koz-3       DGIIQCFIKRDRSTQTYYLYLSLSSAVLVENGKFLLSAKRICRATWTEYIISMNANSISS    180
            .***********************************************************

Bd21        SSNTYLGKMRSNVIGTKFIMYDTQPPYNSSTVSNSGKPSQRFYSKNGSTKVPCSLYSIAQ    240
Bd30-1      SSNTYLGKMRSNVIGTKFIMHDTQPPYNSSTVSHSGKPSQRFYSKNGPTKVPCSLYSIAQ    240
RON2        SSNTYLGKMRSNVIGTKFIMYDTQPPYNSSTVSHSGKPSQRFYSKNGPTKVPCSLYSIAQ    240
Bd21-3      SSNTYLGKMRSNVIGTKFIMYDPQPPYNSSTVSHSGKPSQRFYSKNGSTKVPCSLYSIAQ    240
ABR6        SSNTYLGKMRSNVIGTKFIMYDTQPPYNSSTVSHSGKPSQRFYSKNGSTKVPCSLYSIAQ    240
Bd3-1       SSNTYLGKMRSNVIGTKFIMYDTQPPYNSSTVSHSGKPSQRFYSKNGSTKVPCSLYSIAQ    240
Bd1-1       SSNTYLGKMRSNVIGTKFIMYDTQPPYNSSTVSHSGKPSQRFYSKNGSTKVPCSLYSIAQ    240
Bd2-3       SSNTYLGKMRSNVIGTKFIMYDTQPPYNSSTVSHSGKPSQRFYSKNGSTKVPCSLYSIAQ    240
Bd29-1      SSNTYLGKMRSNVIGTKFIMYDTQPPYNSSTVSHSGKPSQRFYSKNGSTKVPCSLYSIAQ    240
Tek-4       SSNTYLGKMRSNVIGTKFIMYDTQPPYNSSTVSHSGKPSQRFYSKNGSTKVPCSLYSIAQ    240
Koz-3       SSNTYLGKMRSNVIGTKFIMYDTQPPYNSSTVSHSGKPSQRFYSKNGSTKVPCSLYSIAQ    240
            ********************:* **********:************* ************
```

```
Bd21        VSYKLNVLGTRGPRRMHCVMHSIPASSLEAGGTVPCQMDSILARRSLDESFSRISISKSS    300
Bd30-1      VSYKLNVLGTRGPRRMHCVMHSIPASSLEAGGTVPCQMDSILARRSLDESFSRISISKSS    300
RON2        VSYKLNVLGTRGPRRMHCVMHSIPASSLEAGGTVPCQMDSILARRSLDESFSRISISKSS    300
Bd21-3      VSYKLNVLGTRGPRRMHCVMHSIPASSLEAGGTVPCQMDSILARRSLDESFSRISISKSS    300
ABR6        VSYKLNVLGTRGPRRMHCVMHSIPASSLEAGGTVPCQMDSILARRSLDESFSRISISKSS    300
Bd3-1       VSYKLNVLGTRGPRRMHCVMHSIPASSLEAGGTVPCQMDSILARRSLDESFSRISISKSS    300
Bd1-1       VSYKLNVLGTRGPRRMHCVMHSIPASSLEAGGTVPCQMDSILARRSLDESFSRISISKSS    300
Bd2-3       VSYKLNVLGTRGPRRMHCVMHSIPASSLEAGGTVPCQMDSILARRSLDESFSRISISKSS    300
Bd29-1      VSYKLNVLGTRGPRRMHCVMHSIPASSLEAGGTVPCQMDSILARRSLDESFSRISISKSS    300
Tek-4       VSYKLNVLGTRGPRRMHCVMHSIPASSLEAGGTVPCQMDSILARRSLDESFSRISISKSS    300
Koz-3       VSYKLNVLGTRGPRRMHCVMHSIPASSLEAGGTVPCQMDSILARRSLDESFSRISISKSS    300
            ************************************************************

Bd21        IMDHSVHFSSARFSDSSIRGGPGISRQSLSDEDQCKDVPLILRNKAPRWHEQLQCWCLNF    360
Bd30-1      IMDHSMHFSSARFSDSSIRGGPGISGQSLSDEDQCKDVPLILRNKAPRWHEQLQCWCLNF    360
RON2        IMDHSMHFSSARFSDSSIRGGPGISGQSLSDEDQCKDVPLILRNKAPRWHEQLQCWCLNF    360
Bd21-3      IMDHSMHFSSARFSDSSIRGGPGISGQSLSDEDQCKDVPLILRNKAPRWHEQLQCWCLNF    360
ABR6        IMDHSMHFSSARFSDSSIRGGPGISGQSLSDEDQCKDVPLILRNKAPRWHEQLQCWCLNF    360
Bd3-1       IMDHSMHFSSARFSDSSIRGGPGISGQSLSDEDQCKDVPLILRNKAPRWHEQLQCWCLNF    360
Bd1-1       IMDHSMHFSSARFSDSSIRGGPGISGQSLSDEDQCKDVPLILRNKAPRWHEQLQCWCLNF    360
Bd2-3       IMDHSMHFSSARFSDSSIRGGPGISGQSLSDEDQCKDVPLILRNKAPRWHEQLQCWCLNF    360
Bd29-1      IMDHSMHFSSARFSDSSIRGGPGISGQSLSDEDQCKDVPLILRNKAPRWHEQLQCWCLNF    360
Tek-4       IMDHSMHFSSARFSDSSIRGGPGISGQSLSDEDQCKDVPLILRNKAPRWHEQLQCWCLNF    360
Koz-3       IMDHSMHFSSARFSDSSIRGGPGISGQSLSDEDQCKDVPLILRNKAPRWHEQLQCWCLNF    360
            *****:******************* **********************************

Bd21        RGRVTVASVKNFQLTAAAALPAAGALTPPHPAEPAPPPPPEHDKVILQFGKVAKDMFTMD    420
Bd30-1      RGRVTVASVKNFQLTAAAALPAAGALTPPHPAEPAPPPPPEHDKVILQFGKVAKDMFTMD    420
RON2        RGRVTVASVKNFQLTAAAALPAAGALTPPHPAEPAPPPPPEHDKVILQFGKVAKDMFTMD    420
Bd21-3      RGRVTVASVKNFQLTAAAALPAAGALTPPHPAEPAPPPPPEHDKVILQFGKVAKDMFTMD    420
ABR6        RGRVTVASVKNFQLTAAAALPAAGALTPPHPAEPAPPPPPEHDKVILQFGKVAKDMFTMD    420
Bd3-1       RGRVTVASVKNFQLTAAAALPAAGALTPPHPAEPAPPPPPEHDKVILQFGKVAKDMFTMD    420
Bd1-1       RGRVTVASVKNFQLTAAAALPAAGALTPPHPAEPAPPPPPEHDKVILQFGKVAKDMFTMD    420
Bd2-3       RGRVTVASVKNFQLTAAAALPAAGALTPPHPAEPAPPPPPEHDKVILQFGKVAKDMFTMD    420
Bd29-1      RGRVTVASVKNFQLTAAAALPAAGALTPPHPAEPAPPPPPEHDKVILQFGKVAKDMFTMD    420
Tek-4       RGRVTVASVKNFQLTAAAALPAAGPLTPPPPAEPAPPPPPEHDKVILQFGKVAKDMFTMD    420
Koz-3       RGRVTVASVKNFQLTAAAALPAAGALTPPPPAEPAPPPPPEHDKVILQFGKVAKDMFTMD    420
            ************************ **** ******************************

Bd21        YRYPLSAFQAFAISLSCFNTKWGCE   445
Bd30-1      YRYPLSAFQAFAISLSCFNTKWGCE   445
RON2        YRYPLSAFQAFAISLSCFNTKWGCE   445
Bd21-3      YCYPLPAFQAFAISLSCFNTKWGCE   445
ABR6        YRYPLSAFQAFAISLSCFNTKWGCE   445
Bd3-1       YCYPLSAFQAFAISLSCFNTKWGCE   445
Bd1-1       YCYPLSAFQAFAISLSCFNTKWGCE   445
Bd2-3       YCYPLSAFQAFAISLSCFNTKWGCE   445
Bd29-1      YCYPLSAFQAFAISLSCFNTKWGCE   445
Tek-4       YCYPLSAFQAFAISLSCFNTKWGCE   445
Koz-3       YCYPLSAFQAFAISLSCFNTKWGCE   445
            * *** *******************
```

BdTLP7

```
Bd21-3      MSWRSMIREMKGEVGAMSLRGLRSRPSSGRDRRVAPDEAARQSSCWAQLPPELLREVLGG        60
Koz-3       MSLRSMIREMKGEVGAMSLRGLRSRPSSGRDRRVGPGEAARQSSCWAQLPPELLREVLGR        60
Bd1-1       MSLRSMIREMKGEVGAMSLRGLRSRPSSGRDRRVAPDEAARQSSCWAQLPPELLREGLGG        60
Bd29-1      MSLRSMIREMKGEVGAMSLRGLRSRPSSGRDRRVAPDEAARQSSCWAQLPPELLREVLGR        60
Bd30-1      MSLRSMIREMKGEVGAMSLRGLRSRPSSGRDRRVAPDEAARQSSCWAQLPPELLREVLGR        60
Tek-4       ------------------------------------------------------------        0
Bd3-1       ------------------------------------------------------------        0
Bd21        MSLRSMIREMKGEVGAMSLRGLRSRPSSGRDRRVAPDEAARQSSCWAQLPPELLREVLGR        60
ABR6        MSLRSMIREMKGEVGAMSLRGLRSRPSSGRDRRVAPDEAARQSSCWAQLPPELLREVLGR        60
Bd2-3       MSLRSMIREMKGEVGAMSLRGLRSRPSSGRDRRVAPDEAARQSSCWAQLPPELLREVLGR        60
RON2        MSLRSMIREMKGEVGAMSLRGLRSRPSSGRDRRVAPDEAARQSSCWAQLPPELLREVLGR        60

Bd21-3      VEESEPRWPRRRDVVACAGVCQSWRGAVREIVRVPEESGKLTFPISLKQPGPRDAPIKCF        120
Koz-3       VEESEPRWPRRRDVVACAGVCQSWRGAVREIVRVPEESGKLTFPISLKQPGPRDAPIKCF        120
Bd1-1       VEESEPRGPRRRDVVACAGVCQSWRGAVTEIVRVPEESGKLTFPISLKQPGPRDAPIKCF        120
Bd29-1      VEESEPRWPRRRDVVACAGVCQSWRGAVTEIVRVPEESGKLTFPISLKQPGPRDAPIKCF        120
Bd30-1      VEESEPRWPRRRDVVACAGVCQSWRGAVREIVRVPEESGKLTFPISLKQPGPRDAPIKCF        120
Tek-4       ------------------------------------------------------------        0
Bd3-1       ------------------------------------------------------------        0
Bd21        VEESEPRWPRRRDVVACAGVCQSWRGAVREIVRVPEESGKLTFPISLKQPGPRDAPIKCF        120
ABR6        VEESEPRWPRRRDVVACAGVCQSWRGAVREIVRVPEESGKLTFPISLKQPGPRDAPIKCF        120
Bd2-3       VEESEPRWPRRRDVVACAGVCQSWRGAVREIVRVPEESGKLTFPISLKQPGPRDAPIKCF        120
RON2        VEESEPRWPRRRDVVACAGVCQSWRGAVREIVRVPEESGKLTFPISLKQPGPRDAPIKCF        120

Bd21-3      IRRNRATHTYCLYI---GLTNALADDGKFLLAARKYHRPTCTEYLISLDRTDMSKGSQTC        177
Koz-3       IRRNRATHTYCLYI---GLTNALADDGKFLLAARKYHRPTCTEYLISLDRTDMSKGSQTC        177
Bd1-1       IRRNRATHTYCLYI---GLTNALADDGKFLLAARKYHRPTCTEYLISLDRTDMSKGSQTC        177
Bd29-1      IRRNRATHTYCLYI---GLTNALADDGKFLLAARKYHRPTCTEYLISLDRTDMSKGSQTC        177
Bd30-1      IRRNRATHTYCLYI---GLTDALADDGKFLLAARKYHRPTCTEYLILLDRTDMSKGSQTC        177
Tek-4       ---------MCLFYTSNVLFAALADDGKFLLAARKYHRPTCTEYLISLDRTDMSKGSQTC        51
Bd3-1       ----------------------------------------------------MSKGSQTC        8
Bd21        IRRNRATHTYCLYI---GLTNALADDGKFLLAARKYHRPTCTEYLISLDRTDMSKGSQTC        177
ABR6        IRRNRATHTYCLYI---GLTNALADDGKFLLAARKYHRPTCTEYLISLDRTDMSKGSQTC        177
Bd2-3       IRRNRATHTYCLYI---GLTNALADDGKFLLAARKYHRPTCTEYLISLDRTDMSKGSQTC        177
RON2        IRRNRATHTYCLYI---GLTNALADDGKFLLAARKYHRPTCTEYLISLDRTDMSKGSQTC        177
                                                                ********

Bd21-3      IGKLRSNFLRTKFTVYDAHPPHSGAVVSKRWSSGNYPVSHIYYDVNVLGSRGPRRMRCVM        237
Koz-3       IGKLRSNFLRTKFTVYDAHPPHSGAVVSKRWSSGNYPVSHIYYDVNVLGSRGPRRMRCVM        237
Bd1-1       IGKLRSNFLRTKFTVYDAHPPHSGAVVSKRWSSGNYPVSHIYYDVNVLGSRGPRRMRCVM        237
Bd29-1      IGKLRSNFLRTKFTVYDAHPPHSGAVVSKRWSSGNYPVSHIYYDVNVLGSRGPRRMRCVM        237
Bd30-1      IGKLRSNFLRTKFTVYDAHPPHSGAVVSKRWSSGNYPVSHIYYDVNVLGSRGPRRMRCVM        237
Tek-4       IGKLRSNFLRTKFTVYDAHPPHSGAVVSKRWSSGNYPVSHIYYDVNVLGSRGPRRMRCVM        111
Bd3-1       IGKLRSNFLRTKFTVYDAHPPHSGAVVSKRWSSGNYPVSHIYYDVNVLGSRGPRRMRCVM        68
Bd21        IGKLRSNFLRTKFTVYDAHPPHSGAVVSKRWSSGNYPVSHIYYDVNVLGSRGPRRMRCVM        237
ABR6        IGKLRSNFLRTKFTVYDAHPPHSGAVVSKRWSSGNYPVSHIYYDVNVLGSRGPRRMRCVM        237
Bd2-3       IGKLRSNFLRTKFTVYDAHPPHSGAVVSKRWSSGNYPVSHIYYDVNVLGSRGPRRMRCVM        237
RON2        IGKLRSNFLRTKFTVYDAHPPHSGAVVSKRWSSGNYPVSHIYYDVNVLGSRGPRRMRCVM        237
            ************************************************************
```

```
Bd21-3      DSIPVSAIKGGTAPTQTGFPSSSHDSFASIPFFKSKSSRRDSSTTQLSTQEESKLVLKNK        297
Koz-3       DSIPVSAIKGGTAPTQTGFPSSSHDSFASIPFFKSKSSRRDSSTTQLSTQEESKLVLKNK        297
Bd1-1       DSIPVSAIKGGTAPTQTGFPSSSHDSFASIPFFKSKSSRRDSSTTQLSTQEESKLVLKNK        297
Bd29-1      DSIPVSAIKGGTAPTQTGFPSSSHDSFASIPFFKSKSSRRDSSTTQLSTQEESKLVLKNK        297
Bd30-1      DSIPVSAIKGGTAPTQTGFPSSSHDSFASIPFFKSKSSRRDSSTTQLSTQEESKLVLKNK        297
Tek-4       DSIPVSAIKGGTAPTQTGFPSSSHDSFASIPFFKSKSSRRDSSTTQLSTQEESKLVLKNK        171
Bd3-1       DSIPVSAIKGGTAPTQTGFPSSSHDSFASIPFFKSKSSRRDSSTTQLSTQEESKLVLKNK        128
Bd21        DSIPVSAIKGGTAPTQTGFPSSSHDSFASIPFFKSKSSRRDSSTTQLSTQEESKLVLKNK        297
ABR6        DSIPVSAIKGGTAPTQTGFPSSSHDSFASIPFFKSKSSRRDSSTTQLSTQEESKLVLKNK        297
Bd2-3       DSIPVSAIKGGTAPTQTGFPSSSHDSFASIPFFKSKSSRRDSSTTQLSTQEESKLVLKNK        297
RON2        DSIPVSAIKGGTAPTQTGFPSSSHDSFASIPFFKSKSSRRDSSTTQLSTQEESKLVLKNK        297
            ************************************************************

Bd21-3      SPRWQEHLQCWCLNFHGRVTVASVKNFQLVPSDESAPTNHELDDATLQFGKIGKDMFSMD        357
Koz-3       SPRWQEHLQCWCLNFHGRVTVASVKNFQLVPSDESAPTNHELDDATLQFGKIGKDMFSMD        357
Bd1-1       SPRWQEHLQCWCLNFHGRVTVASVKNFQLVPSDESAPTNHELDDATLQFGKIGKDMFSMD        357
Bd29-1      SPRWQEHLQCWCLNFHGRVTVASVKNFQLVPSDESAPTNHELDDATLQFGKIGKDMFSMD        357
Bd30-1      SPRWQEHLQCWCLNFHGRVTVASVKNFQLVPSDESAPTNHELDDATLQFGKIGKDMFSMD        357
Tek-4       SPRWQEHLQCWCLNFHGRVTVASVKNFQLVPSDESAPTNHELDDATLQFGKIGKDMFSMD        231
Bd3-1       SPRWQEHLQCWCLNFHGRVTVASVKNFQLVPSDESAPTNHELDDATLQFGKIGKDMFSMD        188
Bd21        SPRWQEHLQCWCLNFHGRVTVASVKNFQLVPSDESAPTNHELDDATLQFGKIGKDMFSMD        357
ABR6        SPRWQEHLQCWCLNFHGRVTVASVKNFQLVPSDESAPTNHELDDATLQFGKIGKDMFSMD        357
Bd2-3       SPRWQEHLQCWCLNFHGRVTVASVKNFQLVPSDESAPTNHELDDATLQFGKIGKDMFSMD        357
RON2        SPRWQEHLQCWCLNFHGRVTVASVKNFQLVPSDESAPTNHELDDATLQFGKIGKDMFSMD        357
            ************************************************************

Bd21-3      YRYPLSAFQAFAICLSSFDTKIACE   382
Koz-3       YRYPLSAFQAFAICLSSFDTKIACE   382
Bd1-1       YRYPLSAFQAFAICLSSFDTKIACE   382
Bd29-1      YRYPLSAFQAFAICLSSFDTKIACE   382
Bd30-1      YRYPLSAFQAFAICLSSFDTKIACE   382
Tek-4       YRYPLSAFQAFAICLSSFDTKIACE   256
Bd3-1       YRYPLSAFQAFAICLSSFDTKIACE   213
Bd21        YRYPLSAFQAFAICLSSFDTKIACE   382
ABR6        YRYPLSAFQAFAICLSSFDTKIACE   382
Bd2-3       YRYPLSAFQAFAICLSSFDTKIACE   382
RON2        YRYPLSAFQAFAICLSSFDTKIACE   382
            *************************
```

BdTLP8

```
Bd3-1     ------------------------------------RGGSRHSWPGLWPEPQSQPQRQGQ   24
Bd1-1     MSLKSIVRELKEMRDGIGSMSRRAGGSDGRVGPGHSRGGARHSWPGLGPEPQSQPQRRGR   60
Koz-3     MSLKSIVRELKEMRDGIGSMSRRAGGSDGRVGHGPSRGGARHSWPGLGREPQSQPQRQGQ   60
Bd30-1    MSLKSIVRELKEMRDGIGSMSRRAGGSDGRGGHGHSRGGSRHSWPGLWREPQSQPQRQGQ   60
Tek-4     MSLKSIVRELKEMRDGIGSMSRRAGGSDGRVGHGHSRGGSRHSWPGLWPEPQSQPQRQGQ   60
Bd29-1    MSLKSIVRELKEMRDGIGSMSRRAGGSDGRVGHGHSRGGSRHSWPGLWPEPQSQPQRQGQ   60
Bd21-3    MSLKSIVRELKEMRDGIGSMSRRAGGSDGRVGHGHSRGGSRHSWPGLWPEPQSQPQRQGQ   60
Bd21      MSLKSIVRELKEMRDGIGSMSRRAGGSDGRVGHGHSRGGSRHSWPGLWPEPQSQPQRQGQ   60
ABR6      MSLKSIVRELKEMRDGIGSMSRRAGGSDGRVGHGHSRGGSRHSWPGLWPEPQSQPQRQGQ   60
RON2      MSLKSIVRELKEMRDGIGSMSRRAGGSDGRVGHGHSRGGSRHSWPGLWPEPQSQPQRQGQ   60
Bd2-3     MSLKSIVRELKEMRDGIGSMSRRAGGSDGRVGPGHSRGGSRHSWPGLWPEPQSQPQRQGQ   60
                                                ***:*******  ********:*:

Bd3-1     EPQQQGQWANLPPELLLDVIQRVEASEAVWPSRRPGGGGAAVCRSWREVTKDVVKTLEEC   84
Bd1-1     EPQQQGQWANLPPELLLDVIQRVEASEAVWPSRRQVVACAAVCRSWREVTKDVVKTLEEC   120
Koz-3     EPQQQGQWANLPPELLLDVIQRVEASEAVWPSRRQVVACAAVCRSWREVTKDVVKTLEEC   120
Bd30-1    EPQQQGQWANLPPELLLDVIQRVEASEAVWPSRRQGVACAAVCRSWREVTKDVVKTLEEC   120
Tek-4     EPQQQGQWANLPPELLLDVIQRVEASEAVWPSRRQVVACAAVCRSWREVTKDVVKTLEEC   120
Bd29-1    EPQQQGQWANLPPELLLDVIQRVEASEAVWPSRRQVVACAAVCRSWREVTKDVVKTLEEC   120
Bd21-3    EPQQQGQWANLPPELLLDVIQRVEASEAVWPSRRQVVACAAVCRSWREVTKDVVKTLEEC   120
Bd21      EPQQQGQWANLPPELLLDVIQRVEASEAVWPSRRQVVACAAVCRSWREVTKDVVKTLEEC   120
ABR6      EPQQQGQWANLPPELLLDVIQRVEASEAVWPSRRQVVACAAVCRSWREVTKDVVKTLEEC   120
RON2      EPQQQGQWANLPPELLLDVIQRVEASEAVWPSRRQVVACAAVCRSWREVTKDVVKTLEEC   120
Bd2-3     EPQQQGQWANLPPELLLDVIQRVEASEAVWPSRRQVVACAAVCRSWREVTKDVVKTLEEC   120
          **********************************   . *********************

Bd3-1     GRITFPISLKQPGPRDHPVQCFVRRDRATSTYLLYLGLSPSLHVENDKLLLAARKIRRAT   144
Bd1-1     GRITFPISLKQPGPRDHPVQCFVRRDRATSTYLLYLGLSPSLHGENDKLLLAARKIRRAT   180
Koz-3     GRITFPISLKQPGPRDHPVQCFVRRDRATSTYLLYLGLSPSLHVENDKLLLAARKIRRAT   180
Bd30-1    GRVTFPISLKQPGPRDHPVQCFVRRDRATSTYLLYLGLSPSLHVENDKLLLAARKIRRAT   180
Tek-4     GRITFPISLKQPGPRDHPVQCFVRRDRATSTYLLYLGLSPSLHGENDKLLLAARKIRRAT   180
Bd29-1    GRITFPISLKQPGPRDHPVQCFVRRDRATSTYLLYLGLSPSLHGENDKLLLAARKIRRAT   180
Bd21-3    GRVTLPISLKQPGPRDHPVQCFVRRDRATSTYLLYLGLSPSLHVENDKLLLAARKIRRAT   180
Bd21      GRITFPISLKQPGPRDHPVQCFVRRDRATSTYLLYLGLSPSLHVENDKLLLAARKIRRAT   180
ABR6      GRITFPISLKQPGPRDHPVQCFVRRDRATSTYLLYLGLSPSLHVENDKLLLAARKIRRAT   180
RON2      GRITFPISLKQPGPRDHPVQCFVRRDRATSTYLLYLGLSPSLHVENDKLLLAARKIRRAT   180
Bd2-3     GRITFPISLKQPGPRDHPVQCFVRRDRATSTYLLYLGLSPSLHVENDKLLLAARKIRRAT   180
          **:*:************************************** ****************

Bd3-1     RTSFVISLISDDFSHSSSTYVGKLKPNFLGTKFTIIDSQPPCDAAVLPNNRPSKRHSKQV   204
Bd1-1     RTSFVISLISDDFSHSSSTYVGKLKPNFLGTKFTIIDSQPPCDAAVLPNNRPSKRHSKQV   240
Koz-3     RTSFVISLISDDFSHSSSTYVGKLKPNFLGTKFTIIDSQPPCDAAVLPNNRPSKRHSKQV   240
Bd30-1    RTSFVISLISDDFSHSSSTYVGKLKPNFLGTKFTIIDSQPPCDAAVLPNNRPSKRHSKQV   240
Tek-4     RTSFVISLISDDFSHSSSTYVGKLKPNFLGTKFTIIDSQPPCDAAVLPNNRPSKRHSKQV   240
Bd29-1    RTSFVISLISDDFSHSSSTYVGKLKPNFLGTKFTIIDSQPPCDAAVLPNNRPSKRHSKQV   240
Bd21-3    RTSFVISLISDDFSHSSSTYVGKLKPNFLGTKFTIIDSQPPCDAAVLPNNRPSKRHSKQV   240
Bd21      RTSFVISLISDDFSHSSSTYVGKLKPNFLGTKFTIIDSQPPCDAAVLPNNRPSKRHSKQV   240
ABR6      RTSFVISLISDDFSHSSSTYVGKLKPNFLGTKFTIIDSQPPCDAAVLPNNRPSKRHSKQV   240
RON2      RTSFVISLISDDFSHSSSTYVGKLKPNFLGTKFTIIDSQPPCDAAVLPNNRPSKRHSKQV   240
Bd2-3     RTSFVISLISDDFSHSSSTYVGKLKPNFLGTKFTIIDSQPPCDAAVLPNNRPSKRHSKQV   240
          ************************************************************
```

```
Bd3-1       SPRLPLGNYNVATVSYELTVLRNRGPRRMQCTMHSIPALCIQEGGKAPTPTGIIHSLDDQ        264
Bd1-1       SPRLPLGNYNVATVSYELTVLRNRGPRRMQCTMHSIPALCIQEGGKAPTPTGIIHSLDDQ        300
Koz-3       SPRLPLGNYNVATVSYELTVLRNRGPRRMQCTMHSIPALCIQEGGKAPTPTGIIHSLDDQ        300
Bd30-1      SPRLPLGNYNVATVSYELTVLRNRGPRRMQCTMHSIPALCIQEGGKAPTPTGIIHSLDDQ        300
Tek-4       SPRLPLGNYNVATVSYELTVLRNRGPRRMQCTMHSIPALCIQEGGKAPTPTGIIHSLDDQ        300
Bd29-1      SPRLPLGNYNVATVSYELTVLRNRGPRRMQCTMHSIPALCIQEGGKAPTPTGIIHSLDDQ        300
Bd21-3      SPRLPLGNYNVATVSYELTVLRNRGPRRMQCTMHSIPALCIQEGGKAPTPTGIIHSLDDQ        300
Bd21        SPRLPLGNYNVATVSYELTVLRNRGPRRMQCTMHSIPALCIQEGGKAPTPTGIIHSLDDQ        300
ABR6        SPRLPLGNYNVATVSYELTVLRNRGPRRMQCTMHSIPALCIQEGGKAPTPTGIIHSLDDQ        300
RON2        SPRLPLGNYNVATVSYELTVLRNRGPRRMQCTMHSIPALCIQEGGKAPTPTGIIHSLDDQ        300
Bd2-3       SPRLPLGNYNVATVSYELTVLRNRGPRRMQCTMHSIPALCIQEGGKAPTPTGIIHSLDDQ        300
            ************************************************************

Bd3-1       VSTLSTSKGKEPNVEFSSTSLSTDLSGQICTSEAPMLLKNKAPRWHEQLQCWCLNFRGRV        324
Bd1-1       VSTLSTSKGKEPNVEFSSTSLSADLSGQICTSEAPMLLKNKAPRWHEQLQCWCLNFRGRV        360
Koz-3       VSTLSTSKGKEPNVEFSSTSLSTDLSGQICTSEAPMLLKNKAPRWHEQLQCWCLNFRGRV        360
Bd30-1      VSTLSTSKGKEPNVEFSSTSLSTDLSGQICTSEAPMLLKNKAPRWHEQLQCWCLNFRGRV        360
Tek-4       VSTLSTSKGKEPNVEFSSTSLSADLSGQICTSEAPMLLKNKAPRWHEQLQCWCLIFRGRV        360
Bd29-1      VSTLSTSKGKEPNVEFSSTSLSADLSGQICTSEAPMLLKNKAPRWHEQLQCWCLNFRGRV        360
Bd21-3      VSTLSTSKGKEPNVEFSSTSLSTDLSGQICTSEAPMLLKNKAPRWHEQLQCWCLNFRGRV        360
Bd21        VSTLSTSKGKEPNVEFSSTSLSTDLSGQICTSEAPMLLKNKAPRWHEQLQCWCLNFRGRV        360
ABR6        VSTLSTSKGKEPNVEFSSTSLSTDLSGQICTSEAPMLLKNKAPRWHEQLQCWCLNFRGRV        360
RON2        VSTLSTSKGKEPNVEFSSTSLSTDLSGQICTSEAPMLLKNKAPRWHEQLQCWCLNFRGRV        360
Bd2-3       VSTLSTSKGKEPNVEFSSTSLSTDLSGQICTSEAPMLLKNKAPRWHEQLQCWCLNFRGRV        360
            **********************:******************************* *****

Bd3-1       TVASVKNFQLVASVDPSLGVPAAEQEKVILQFGKIGKDIFTMDYRYPLSAFQAFAICLTS        384
Bd1-1       TVASVKNFQLVASVDPSLGVPAAEQEKVILQFGKIGKDIFTMDYRYPLSAFQAFAICLTS        420
Koz-3       TVASVKNFQLVASVDPSLGVPAAEQEKVILQFGKIGKDIFTMDYRYPLSAFQAFAICLTS        420
Bd30-1      TVASVKNFQLVASVDPSLGVPAAEQEKVILQFGKIGKDIFTMDYRYPLSAFQAFAICLTS        420
Tek-4       TVASVKNFQLVASVDPSLGVPAAEQEKVILQFGKIGKDIFTMDYRYPLSAFQAFAICLTS        420
Bd29-1      TVASVKNFQLVASVDPSLGVPAAEQEKVILQFGKIGKDIFTMDYRYPLSAFQAFAICLTS        420
Bd21-3      TVASVKNFQLVASVDPSLGVPAAEQEKVILQFGKIGKDIFTMDYRYPLSAFQAFAICLTS        420
Bd21        TVASVKNFQLVASVDPSLGVPAAEQEKVILQFGKIGKDIFTMDYRYPLSAFQAFAICLTS        420
ABR6        TVASVKNFQLVASVDPSLGVPAAEQEKVILQFGKIGKDIFTMDYRYPLSAFQAFAICLTS        420
RON2        TVASVKNFQLVASVDPSLGVPAAEQEKVILQFGKIGKDIFTMDYRYPLSAFQAFAICLTS        420
Bd2-3       TVASVKNFQLVASVDPSLGVPAAEQEKVILQFGKIGKDIFTMDYRYPLSAFQAFAICLTS        420
            ************************************************************

Bd3-1       FDTKPACE    392
Bd1-1       FDTKPACE    428
Koz-3       FDTKPACE    428
Bd30-1      FDTKPACE    428
Tek-4       FDTKPACE    428
Bd29-1      FDTKPACE    428
Bd21-3      FDTKPACE    428
Bd21        FDTKPACE    428
ABR6        FDTKPACE    428
RON2        FDTKPACE    428
Bd2-3       FDTKPACE    428
            ********
```

BdTLP9

```
Bd21-3      ------------------------------------------------------------      0
Bd30-1      MPREKPAAERGEVPEGGGGEEKAEEGGQAE----AEEQEERWARLLPELLSEVVRRVEAS      56
Koz-3       MPREKPAAERGEVPEVVEVEEKAEEGGQAE----AEEQEERWARLLPELLSEVVRRVEAS      56
Bd1-1       MPREKPAAERGEVPEGGEGEEKAEEGGQAEPEAEAEEQEERWARLLPELLSEGGRRVEAS      60
RON2        MPREKPAAERGEVPEVVEVEEKAEEGGQAE----AEEQEERWARLLPELLSEVVRRVEAS      56
Bd2-3       MPREKPAAERGEVPEVVEVEEKAEEGGQAE----AEEQEERGGRLLPELLSEVVRRVEAS      56
Bd29-1      MPREKPAAERGEVPEVVEVEEKAEEGGQAEPEAEAEEQEERWARLLPELLSEVVRRVEAS      60
ABR6        MPREKPAAERGEVPEVVEVEEKAEEGGQAE----AEEQEERWARLLPELLSEVVRRVEAS      56
Bd21        MPREKPAAERGEVPEVVEVEEKAEEGGQAE----AEEQEERWARLLPELLSEVVRRVEAS      56
Bd3-1       ------------------------------------------------------------      0
Tek-4       ------------------------------------------------------------      0


Bd21-3      -----------------------------------------------------MQCFIKR      7
Bd30-1      GGERWPARKDVVSCACVCRRGRDAAVAVGRPPAVSGKITFPSSLKQPGTREFPMQCFIKR      116
Koz-3       GGERWPARKDVVSCACVCRRGRDAAVAVVRPPAVSGKITFPSSLKQPGTREFPMQCFIKR      116
Bd1-1       GGERWPARKDVVSCACVCRRGRDAAVAVVRPPAVSGKITFPSSLKQPGTREFPMQCFIKR      120
RON2        GGERWPARKDVVSCACVCRRWRDAAVAVVRPPAVSGKITFPSSLKQPGTREFPMQCFIKR      116
Bd2-3       GGERWPARKDVVSCACVCRRWRDAAVAVVRPPAVSGKITFPSSLKQPGTREFPMQCFIKR      116
Bd29-1      GGERWPARKDVVSCACVCRRWRDAAVAVVRPPAVSGKITFPSSLKQPGTREFPMQCFIKR      120
ABR6        GGERWPARKDVVSCACVCRRWRDAAVAVVRPPAVSGKITFPSSLKQPGTREFPMQCFIKR      116
Bd21        GGERWPARKDVVSCACVCRRWRDAAVAVVRPPAVSGKITFPSSLKQPGTREFPMQCFIKR      116
Bd3-1       --------MDVVSCACVCRRWRDAAGAAVRPPAGSGKITFPSSLKQPGTREFPMQCFIKR      52
Tek-4       -----------------------------------------------------MQCFIKR      7
                                                                 *******

Bd21-3      NKKNSTFYLYLGLTTATVDKGKFLMAARRFRRGPHTEYIISLDADDLSQGSNAYMGKLRS      67
Bd30-1      NKKNSTFYLYLGLTTATVDKGKFLMAARRFRRGPHTEYIISLDADDLSQGSNAYMGKLRS      176
Koz-3       NKKNSTFYLYLGLTTATVDKGKFLMAARRFRCGPHTEYIISLDADDLSQGSNAYMGKLRS      176
Bd1-1       NKKNSTFYLYLGLTTATVDKGKFLMAARRFRRGPHTEYIISLDADDLSQGSNAYMGKLRS      180
RON2        NKKNSTFYLYLGLTTATVDKGKFLMAARRFRRGPHTEYIISLDADDLSQGSNAYMGKLRS      176
Bd2-3       NKKNSTFYLYLGLTTATVDKGKFLMAARRFRRGPHTEYIISLDADDLSQGSNAYMGKLRS      176
Bd29-1      NKKNSTFYLYLGLTTATVDKGKFLMAARRFRRGPHTEYIISLDADDLSQGSNAYMGKLRS      180
ABR6        NKKNSTFYLYLGLTTATVDKGKFLMAARRFRRGPHTEYIISLDADDLSQGSNAYMGKLRS      176
Bd21        NKKNSTFYLYLGLTTATVDKGKFLMAARRFRRGPHTEYIISLDADDLSQGSNAYMGKLRS      176
Bd3-1       NKKNSTFYLYLGLTTATVDKGKFLMAARRFRRGPHTEYIISLDADDLSQGSNAYMGKLRS      112
Tek-4       NKKNSTFYLYLGLTTATVDKGKFLMAARRFRRGPHTEYIISLDADDLSQGSNAYMGKLRS      67
            ******************************* ****************************

Bd21-3      DFWGTNFKVYDSKPPYDGAKASSSRSSRRFGSRRISPQVSAGNYEVGQLSYKYNLLKSRG      127
Bd30-1      DFWGTNFKVYDSKPPYDGAKASSSRSSRRFGSRRISPQVSAGNYEVGQLSYKYNLLKSRG      236
Koz-3       DFWGTNFKVYDSKPPYDGAKASSSRSSRRFGSRRISPQVSAGNYEVGQLSYKYNLLKSRG      236
Bd1-1       DFWGTNFKVYDSKPPYDGAKASSSRSSRRFGSRRISPQVSAGNYEGGQLSYKYNLLKSRG      240
RON2        DFWGTNFKVYDSKPPYDGAKASSSRSSRRFGSRRISPQVSAGNYEVGQLSYKYNLLKSRG      236
Bd2-3       DFWGTNFKVYDSKPPYDGAKASSSRSSRRFGSRRISPQVSAGNYEVGQLSYKYNLLKSRG      236
Bd29-1      DFWGTNFKVYDSKPPYDGAKASSSRSSRRFGSRRISPQVSAGNYEVGQLSYKYNLLKSRG      240
ABR6        DFWGTNFKVYDSKPPYDGAKASSSRSSRRFGSRRISPQVSAGNYEVGQLSYKYNLLKSRG      236
Bd21        DFWGTNFKVYDSKPPYDGAKASSSRSSRRFGSRRISPQVSAGNYEVGQLSYKYNLLKSRG      236
Bd3-1       DFWGTNFKVYDSKPPYDGAKASSSRSSRRFGSRRISPQVSAGNYEVGQLSYKYNLLKSRG      172
Tek-4       DFWGTNFKVYDSKPPYDGAKASSSRSSRRFGSRRISPQVSAGNYEVGQLSYKYNLLKSRG      127
            ********************************************* **************
```

```
Bd21-3      PRRMYCALECPSTQETWENSLKSKFQKPMGTTVLRNKAPRWHEHLQCWCLNFHGRVTVAS        187
Bd30-1      PRRMYCALECPSTQETWENSLKSKFQKPMGTTVLRNKAPRWHEHLQCWCLNFHGRVTVAS        296
Koz-3       PRRMYCALECPSTQETWENSLKSKFQKPMGTTVLRNKAPRWHEHLQCWCLNFHGRVTVAS        296
Bd1-1       PRRMYCALECPSTQETWENSLKSKFQKPMGTTVLRNKAPRWHEHLQCWCLNFHGRVTVAS        300
RON2        PRRMYCALECPSTQETWENSLKSKFQKPMGTTVLRNKAPRWHEHLQCWCLNFHGRVTVAS        296
Bd2-3       PRRMYCALECPSTQETWENSLKSKFQKPMGTTVLRNKAPRWHEHLQCWCLNFHGRVTVAS        296
Bd29-1      PRRMYCALECPSTQETWENSLKSKFQKPMGTTVLRNKAPRWHEHLQCWCLNFHGRVTVAS        300
ABR6        PRRMYCALECPSTQETWENSLKSKFQKPMGTTVLRNKAPRWHEHLQCWCLNFHGRVTVAS        296
Bd21        PRRMYCALECPSTQETWENSLKSKFQKPMGTTVLRNKAPRWHEHLQCWCLNFHGRVTVAS        296
Bd3-1       PRRMYCALECPSTQETWENSLKSKFQKPMGTTVLRNKAPRWHEHLQCWCLNFHGRVTVAS        232
Tek-4       PRRMYCALECPSTQETWENSLKSKFQKPMGTTVLRNKAPRWHEHLQCWCLNFHGRVTVAS        187
            ************************************************************

Bd21-3      VKNFQLVAAADPNDPTGSRGEDTVLLQFGKVDDDIFTMDYRQPLPAFQAFAISLSSFGTK        247
Bd30-1      VKNFQLVAAADPNDPTGSRDEDTVLLQFGKVDDDIFTMDYRQAPSAFQAFAISLSSFGTK        356
Koz-3       VKNFQLVAAADPNDPTGSRDEDTVLLQFGKVDDDIFTMDYRQPLSAFQAFAISLSSFGTK        356
Bd1-1       VKNFQLVAAADPNDPTGSRDEDTVLLQFGKVDDDIFTMDYRQPLSAFQAFAISLSSFGTK        360
RON2        VKNFQLVAAADPNDPTGSRDEDTVLLQFGKVDDDIFTMDYRQPLSAFQAFAISLSSFGTK        356
Bd2-3       VKNFQLVAAADPNDPTGSRDEDTVLLQFGKVDDDIFTMDYRQPLSAFQAFAISLSSFGTK        356
Bd29-1      VKNFQLVAAADPNDPTGSRDEDTVLLQFGKVDDDIFTMDYRQPLSAFQAFAISLSSFGTK        360
ABR6        VKNFQLVAAADPNDPTGSRDEDTVLLQFGKVDDDIFTMDYRQPLSAFQAFAISLSSFGTK        356
Bd21        VKNFQLVAAADPNDPTGSRDEDTVLLQFGKVDDDIFTMDYRQPLSAFQAFAISLSSFGTK        356
Bd3-1       VKNFQLVAAADPNDPTGSRDEDTVLLQFGKVDDDIFTMDYRQPLSAFQAFAISLSSFGTK        292
Tek-4       VKNFQLVAAADPNDPTGSRDEDTVLLQFGKVDDDIFTMDYRQPLSAFQAFAISLSSFGTK        247
            *******************.**********************   ***************

Bd21-3      LACE        251
Bd30-1      LACE        360
Koz-3       LACE        360
Bd1-1       LACE        364
RON2        LACE        360
Bd2-3       LACE        360
Bd29-1      LACE        364
ABR6        LACE        360
Bd21        LACE        360
Bd3-1       LACE        296
Tek-4       LACE        251
            ****
```

BdTLP10

```
Bd3-1      ------------------------------------------------------------      0
Koz-3      -----------------------------------------------------------M      1
Bd30-1     MDDPRAWGKRQSTEGACGWRRLPVARARPGAGGTRGRAAVAVGGGLSQGRTGEGLSFTAT      60
Bd2-3      -----------------------------------------------------------M      1
Bd21-3     -----------------------------------------------------------M      1
Bd21       -----------------------------------------------------------M      1
ABR6       -----------------------------------------------------------M      1
RON2       -----------------------------------------------------------M      1
Bd1-1      -----------------------------------------------------------M      1
Tek-4      ------------------------------------------------------------      0
Bd29-1     -----------------------------------------------------------M      1


Bd3-1      ------------------------------------------------------------      0
Koz-3      ALWR-----RSYSSRLSCSSRSRSPAGAVVGGNEAKVSPEIAPEEDAGRQEEVDEERWSR      56
Bd30-1     LLYRAHSVASIHPPRLACSSRSRPPAGAVVGGNEAKVSPEIAPEEDAGRQEEVDEERWSR      120
Bd2-3      ALWR-----RSSSSWLSCSSCSRSPAGAVVGGNEAKVSPEIAPEEDAGRQEEGDEERWSR      56
Bd21-3     ALWR-----RSYSSWLSCSSCSRSPAGAVVGGNEAKVSPEIAPEEDAGRQEEVDEERWSR      56
Bd21       ALWR-----RSYSSWLSCSSCSRSPAGAVVGGNEAKVSPEIAPEEDAGRQEEVDEERWSR      56
ABR6       ALWR-----RSYSSWLSCSSCSRSPAGAVVGGNEAKVSPEIAPEEDAGRQEEVDEERWSR      56
RON2       ALWR-----RSYSSWLSCSSCSRSPAGAVVGGNEAKVSPEIAPEEDAGRQEEVDEERWSR      56
Bd1-1      ALWR-----RSYSSRLSCSPRPPPPAGAVGGGNEAKVSPEIAPEEDAGRQEEVDEERWSR      56
Tek-4      ------------------------------------------------------------      0
Bd29-1     ALWR-----RSYSSWLSCSSCSRSPAGAVVGGNEAKVSPEIAPEEDAGRQEEVDEERWSR      56


Bd3-1      -----------------------MDVVSCACVCRRWRDAAGAAVRPPAGSGKITFPSSLK      37
Koz-3      LLPELLTEIVRRVDARADRWPPRRDVVACACVCRRWRDAAVSVVRPPLECGRITFPSSLK      116
Bd30-1     LLPELLTEIVRRVDARADRWPPRRDVVACACVCRRWRDAAVSVVRPPLECGRITFPSSLK      180
Bd2-3      LLPELLTEIVRRVDARADRWPPRRDVVACACVCRRWRDAAVSVVRPPLECGRITFPSSLK      116
Bd21-3     LLPELLTEIVRRVDARADRWPPRRDVVACACVCRRWRDAAVSVVRPPLECGRITFPSSLK      116
Bd21       LLPELLTEIVRRVDARADRWPPRRDVVACACVCRRWRDAAVSVVRPPLECGRITFPSSLK      116
ABR6       LLPELLTEIVRRVDARADRWPPRRDVVACACVCRRWRDAAVSVVRPPLECGRITFPSSLK      116
RON2       LLPELLTEIVRRVDARADRWPPRRDVVACACVCRRWRDAAVSVVRPPLECGRITFPSSLK      116
Bd1-1      LLPELLTEIVRRVDARADRWPPRRDVVACACVCRRGRDAAVSVVRPPLECGRITFPSSLK      116
Tek-4      ------------------------------------------------------------      0
Bd29-1     LLPELLTEIVRRVDARADRWPPRRDVVACACVCRRWRDAAVSVVRPPLECGRITFPSSLK      116


Bd3-1      QPGTREFPMQCFIKRNKKNSTFYLYLGLTTATVDKGKFLMAARRFRRGPHTEYIISLDAD      97
Koz-3      QPGPRDAPMHCFIRRNKNNSTFYLYLSLTQALTDKGMFLLAARRFRHGAHTEYIISYDSD      176
Bd30-1     QPGPRDAPMHCFIRRNKNNSTFYLYLSLTQ------------------------------      210
Bd2-3      QPGPRDAPMHCFIRRNKNNSTFYLYLSLTQALTDKGKFLLAARRFRHGAHTEYIISYDSD      176
Bd21-3     QPGPRDAPMHCFIRRNKNNSTFYLYLSLTQALTDKGKFLLAGRRFRHGAHTEYIISYDSD      176
Bd21       QPGPRDAPMHCFIRRNKNNSTFYLYLSLTQALTDKGKFLLAARRFRHGAHTEYIISYDSD      176
ABR6       QPGPRDAPMHCFIRRNKNNSTFYLYLSLTQALTDKGKFLLAARRFRHGAHTEYIISYDSD      176
RON2       QPGPRDAPMHCFIRRNKNNSTFYLYLSLTQALTDKGKFLLAARRFRHGAHTEYIISYDSD      176
Bd1-1      QPGPRDAPMHCFIRRNKNNSTFYLYLSLTQ-----GKFLLAARRFRHGAHTEYIISYDSD      171
Tek-4      ------------------------------------------------------------      0
Bd29-1     QPGPRDAPMHCFIRRNKNNSTFYLYLSLTQALTDKGKFLLAARRFRHGAHTEYIISYDSD      176
```

```
Bd3-1       DLSQGSNAYMGKLRSDFWGTNFKVYDSKPPYDGAKASSSRSSRRFGSRRISPQVSAGNYE     157
Koz-3       DLYPGSNSGVGKMRSDFLGTKFIIYDSQQPYDGAKLLKSRSSRRFASKQISPHVSGGSFE     236
Bd30-1      ----GSNSGVGKMRSDFLGTKFIIYDSQQPYDGAKLLKSRSSRRFASKQISPHVSGGSFE     266
Bd2-3       DLYPGSNSGVGKMRSDFLGTKFIIYDSQQPYDGAKLLKSRSSRRFASKQISPHVSGGSFE     236
Bd21-3      DLYPGSNSGVGKMRSDFLGTKFIIYDSQQPYDGAKLLKSRSSRRFASKQISPHVSGGSFE     236
Bd21        DLYPGSNSGVGKMRSDFLGTKFIIYDSQQPYDGAKLLKSRSSRRFASKQISPHVSGGSFE     236
ABR6        DLYPGSNSGVGKMRSDFLGTKFIIYDSQQPYDGAKLLKSRSSRRFASKQISPHVSGGSFE     236
RON2        DLYPGSNSGVGKMRSDFLGTKFIIYDSQQPYDGAKLLKSRSSRRFASKQISPHVSGGSFE     236
Bd1-1       DLYPGSNSGVGKMRSDFLGTKFIIYDSQQPFDGAKLLKSRSSRRFASKQISPHVSGGSFE     231
Tek-4       ------------MRSDFLGTKFIIYDSQQPFDGAKLLKSRSSRRFASKQISPHVSGGSFE     48
Bd29-1      DLYPGSNSGVGKMRSDFLGTKFIIYDSQQPFDGAKLLKSRSSRRFASKQISPHVSGGSFE     236
                        :**** **:* :***: *:****  .*******.*::***:**.*.:*

Bd3-1       VGQLSYKYNLLKSRGPRRMYCALECPSTQETWENSLKSKFQKPMGTTVLRNKAPRWHEHL     217
Koz-3       VGQVTYKFNFLKSRGPRRMQCNIQCPVDQAAASDPSKEKPQSTSSSLSLRNKAPRWHEHL     296
Bd30-1      VGQVTYKFNFLKSRGPRRMQCNIQCPVDQAAASDPSKEKPQSTSSSLSLRNKAPRWHEHL     326
Bd2-3       VGQVTYKFNFLKSRGPRRMQCNIQCPVDQAAASDPSKEKPQSTSSSLSLRNKAPRWHEHL     296
Bd21-3      VGQVTYKFNFLKSRGPRRMQCNIQCPVDQAAASDPSKEKPQSTSSSLSLRNKAPRWHEHL     296
Bd21        VGQVTYKFNFLKSRGPRRMQCNIQCPVDQAAASDPSKEKPQSTSSSLSLRNKAPRWHEHL     296
ABR6        VGQVTYKFNFLKSRGPRRMQCNIQCPVDQAAASDPSKEKPQSTSSSLSLRNKAPRWHEHL     296
RON2        VGQVTYKFNFLKSRGPRRMQCNIQCPVDQAAASDPSKEKPQSTSSSLSLRNKAPRWHEHL     296
Bd1-1       VGQVTYKFNFLKSRGPRRMQCNIQCPVDQAAASDPSKEKPQSTSSSLSLRNKAPRWHEHL     291
Tek-4       VGQVTYKFNFLKSRGPRRMQCNIQCPVDQAAASDPSKEKPQSTSSSLSLRNKAPRWHEHL     108
Bd29-1      VGQVTYKFNFLKSRGPRRMQCNIQCPVDQAAASDPSKEKPQSTSSSLSLRNKAPRWHEHL     296
            ***::**:*:********* * ::**  * : .:  *.* *.  .:  ************

Bd3-1       QCWCLNFHGRVTVASVKNFQLVAAADPNDPTGSRDEDTVLLQFGKVDDDIFTMDYRQPLS     277
Koz-3       QCWCLNFHGRVTVASVKNFQLVAPAGTSNPWGVGDEETVILQFGKIEDDAFTMDFRYPLS     356
Bd30-1      QCWCLNFHGRVTVASVKNFQLVAPAGTSNPWGVGDEETVILQFGKIEDDAFTMDFRYPLS     386
Bd2-3       QCWCLNFHGRVTVASVKNFQLVAPAGTSNPWGVGDEETVILQFGKIEDDAFTMDFRYPLS     356
Bd21-3      QCWCLNFHGRVTVASVKNFQLVAPAGTSNPWGVGDEETVILQFGKIEDDAFTMDFRYPLS     356
Bd21        QCWCLNFHGRVTVASVKNFQLVAPAGTSNPWGVGDEETVILQFGKIEDDAFTMDFRYPLS     356
ABR6        QCWCLNFHGRVTVASVKNFQLVAPAGTSNPWGVGDEETVILQFGKIEDDAFTMDFRYPLS     356
RON2        QCWCLNFHGRVTVASVKNFQLVAPAGTSNPWGVGDEETVILQFGKIEDDAFTMDFRYPLS     356
Bd1-1       QCWCLNFHGRVTVASVKNFQLVAPAGTSNPWGVGDEETVILQFGKIEDDAFTMDFRYPLS     351
Tek-4       QCWCLNFHGRVTVASVKNFQLVAPAGTSNPWGVGDEETVILQFGKIEDDAFTMDFRYPLS     168
Bd29-1      QCWCLNFHGRVTVASVKNFQLVAPAGTSNPWGVGDEETVILQFGKIEDDAFTMDFRYPLS     356
            *********************** *. .:* *  **:**:*****::** ****:* ***

Bd3-1       AFQAFAISLSSFGTKLACE 296
Koz-3       AFQAFAICLTSFGTKLACE 375
Bd30-1      AFQAFAICLTSFGTKLACE 405
Bd2-3       AFQAFAICLTSFGTKLACE 375
Bd21-3      AFQAFAICLTSFGTKLACE 375
Bd21        AFQAFAICLTSFGTKLACE 375
ABR6        AFQAFAICLTSFGTKLACE 375
RON2        AFQAFAICLTSFGTKLACE 375
Bd1-1       AFQAFAICLTSFGTKLACE 370
Tek-4       AFQAFAICLTSFGTKLACE 187
Bd29-1      AFQAFAICLTSFGTKLACE 375
            *******.*:*********
```

BdTLP13

```
Bd30-1      MAAATTTPAKREPLRPLSCNAGAAAVAVVRPRGAATASAEKENQGAPNLGPDKEEKVKGA    60
Bd3-1       MAAATTTPAKREPL----------------------------------------------    14
Bd1-1       MAAATTTPAKREPLRPLSCNAGAAAVAVVRPRGAATASAEKENQGAPNLGPDKEEKGKGA    60
Tek-4       MAAATTTPAKREPLRPLSCNAGAAAVAVVRPRGAATASAEKENQGAPNLGPDKEEKVKVA    60
Bd21-3      MAAATTTPAKREPLRPLSCNAGAAAVAVVRPRGAATASAEKENQGAPNLGPDKEEKVKVA    60
Koz-3       MAAATTTPAKREPLRPLSCNAGAAAVAVVRPRGAATASAEKENQGAPNLGPDKEEKVRVA    60
Bd2-3       MAAATTTPAKREPLRPLSCNAGAAAVAVVRPRGAATASAEKENQGAPNLGPDKEEKVKVA    60
Bd21        MAAATTTPAKREPLRPLSCNAGAAAVAVVRPRGAATASAEKENQGAPNLGPDKEEKVKVA    60
ABR6        MAAATTTPAKREPLRPLSCNAGAAAVAVVRPRGAATASAEKENQGAPNLGPDKEEKVKVA    60
RON2        MAAATTTPAKREPLRPLSCNAGAAAVAVVRPRGAATASAEKENQGAPNLGPDKEEKVKVA    60
Bd29-1      MAAATTTPAKREPLRPLSCNAGAAAVAVVRPRGAATASAEKENQGAPNLGPDKEEKVKVA    60
            **************

Bd30-1      PPPPLKPSSLQARMGGEAPLAAAAPAGGGPGFMGPRGGELLPPATPAAAAAASYEAWDPS    120
Bd3-1       ------------------------------------------------------------    14
Bd1-1       PPPPLKPSSLQARMEGEAPLAAAAPAAVGPVFMGPRGRELLPPATPAAAAAASYEAGGLS    120
Tek-4       PPPPLKPSSLQARMEGEAPLAAAAPAAVVPGFMGPRGRELLPPATPAAAAAASYEAWDLS    120
Bd21-3      PPPPLKPSSLQARMEGEAPLAAAAPAAVVPVFMGPRGRELLPPATPAAAAAASYEAWDLS    120
Koz-3       PPPPLKPSSLQARMEGEAPLAAAAPAAGGPVFMGPRGRELLPPATPAAAAAASYEAWDLS    120
Bd2-3       PPPPLKPSSLQARMEGEAPLAAAGPAAVVPVFMGPRGRELLPPATPAAAAAASYEAWDLS    120
Bd21        PPPPLKPSSLQARMEGEAPLAAAAPAAVVPVFMGPRGRELLPPATPAAAAAASYEAWDLS    120
ABR6        PPPPLKPSSLQARMEGEAPLAAAAPAAVVPVFMGPRGRELLPPATPAAAAAASYEAWDLS    120
RON2        PPPPLKPSSLQARMEGEAPLAAAAPAAVVPVFMGPRGRELLPPATPAAAAAASYEAWDLS    120
Bd29-1      PPPPLKPSSLQARMEGEAPLAAAAPAAVVPVFMGPRGRELLPPATPAAAAAASYEAWDLS    120


Bd30-1      DSEAAPAAAWATPPNRALLCRPLPLDVGRGPCVVVREKAAGPRGVALYSLYTNEGQGRRD    180
Bd3-1       --------------------RPL-----SCNAGAAAVAVGRPRGVALYSLYTNGGQGGQD    49
Bd1-1       DSEAAPAASWAPLPNRALLCRPLPLDVGRCTCVVVREKAAGPRGVALYSLYTNEGQGRQD    180
Tek-4       DSEAAPAASWAPLPNRALLCRPLPLDVGRCTCVVVREKAAGPRGVALYSLYTNEGQGRQD    180
Bd21-3      DSEAAPAASWATLPNRALLCRPLPPDVGRCTCVVVREKAAGPRGVALYSLYTNEGQGRQD    180
Koz-3       DSEAAPAASWATLPNRALLCRPLPLDVGRCTCVVVREKAAGPRGVALYSLYTNEGQGRQD    180
Bd2-3       DSEAAPAASWATLPNRALLCRPLPLDVGRCTCVVVREKAAGPRGVALYSLYTNEGQGRQD    180
Bd21        DSEAAPAASWATLPNRALLCRPLPLDVGRCTCVVVREKAAGPRGVALYSLYTNEGQGRQD    180
ABR6        DSEAAPAASWATLPNRALLCRPLPLDVGRCTCVVVREKAAGPRGVALYSLYTNEGQGRQD    180
RON2        DSEAAPAASWATLPNRALLCRPLPLDVGRCTCVVVREKAAGPRGVALYSLYTNEGQGRQD    180
Bd29-1      DSEAAPAASWATLPNRALLCRPLPLDVGRCTCVVVREKAAGPRGVALYSLYTNEGQGRQD    180
                                ***        . ..   .. ************ *** :*

Bd30-1      RKLAGARHRRRRGRSEFIVAQNQDGVFCSSDKNFLGTVGANLVGSKYQIWGQGNRIDELN    240
Bd3-1       RKLAGARHRRRRGRSEFIVAQNQDGVFCSSDKNFLGTVGANLVGSKYQIWGQGNRIDELN    109
Bd1-1       RKLAGARHRRRRGRAEFIVAQNQDGVFCSSDKNFLGTVGANLVGSKYQIWGQGNRIDELN    240
Tek-4       RKLAGARHRRRRGRSEFIVAQNQDGVFCSSDKNFLGTVGANLVGSKYQIWGQGNRIDELN    240
Bd21-3      RKLAVARHRGRRGRSEFIVAQNQDGVFCSSDKNFLGTVGANLVGSKYQIWGQGNRIDELN    240
Koz-3       RKLAVARHRRRRGRSEFIVAQNQDGVFCSSDKNFLGTVGANLVGSKYQIWGQGNRIDELN    240
Bd2-3       RKLAVARHRRRRGRSEFIVAQNQDGVFCSSDKNFLGTVGANLVGSKYQIWGQGNRIDELN    240
Bd21        RKLAVARHRRRRGRSEFIVAQNQDGVFCSSDKNFLGTVGANLVGSKYQIWGQGNRIDELN    240
ABR6        RKLAVARHRRRRGRSEFIVAQNQDGVFCSSDKNFLGTVGANLVGSKYQIWGQGNRIDELN    240
RON2        RKLAVARHRRRRGRSEFIVAQNQDGVFCSSDKNFLGTVGANLVGSKYQIWGQGNRIDELN    240
Bd29-1      RKLAVARHRRRRGRSEFIVAQNQDGVFCSSDKNFLGTVGANLVGSKYQIWGQGNRIDELN    240
            **** **** ****:*********************************************
```

```
Bd30-1      SQSKRLLGVVAFAPTITTLTGSFRSMRAWIPKSQSMQLRTNSSAQIQHIGGLPKDWEEKA      300
Bd3-1       SQSKRLLGVVAFAPTITTLTGSFRSMRAWIPKSQSMQLRTNSSAQIQHIGGLPKDWEEKA      169
Bd1-1       SQSKRLLGVVAFAPTITTLTGSFRSMRAWIPKSQSMQLRTNSSAQIQHIGGLPKDWEEKA      300
Tek-4       SQSKRLLGVVAFAPTITTLTGSFRSMRAWIPKSQSMQLRTNSSAQIQHIGGLPKDWEEKA      300
Bd21-3      SQSKRLLGVVAFAPTITTLTGSFRSMRAWIPKSQSMQLRTNSSAQIQHIGGLPKDWEEKA      300
Koz-3       SQSKRLLGVVAFAPTITTLTGSFRSMRAWIPKSQSMQLRTNSSAQIQHIGGLPKDWEEKA      300
Bd2-3       SQSKRLLGVVAFAPTITTLTGSFRSMRAWIPKSQSMQLRTNSSAQIQHIGGLPKDWEEKA      300
Bd21        SQSKRLLGVVAFAPTITTLTGSFRSMRAWIPKSQSMQLRTNSSAQIQHIGGLPKDWEEKA      300
ABR6        SQSKRLLGVVAFAPTITTLTGSFRSMRAWIPKSQSMQLRTNSSAQIQHIGGLPKDWEEKA      300
RON2        SQSKRLLGVVAFAPTITTLTGSFRSMRAWIPKSQSMQLRTNSSAQIQHIGGLPKDWEEKA      300
Bd29-1      SQSKRLLGVVAFAPTITTLTGSFRSMRAWIPKSQSMQLRTNSSAQIQHIGGLPKDWEEKA      300
            ************************************************************

Bd30-1      SRADKLCSRSPFYNNMTKRYELDFRERVGRMGYKVQTSVKNFQMTLEENGRQTILQLGRV      360
Bd3-1       SRADKLCSRSPFYNNMTKRYELDFRERVGRMGYKVQTSVKNFQMTLEENGRQTILQLGRV      229
Bd1-1       SRADKLCSRSPFYNNMTKRYELDFRERVGRMGYKVQTSVKNFQMTLEENGRRTILQLGRG      360
Tek-4       SRADKLCSRSPFYNNMTKRYELDFRERVGRMGYKVQTSVKNFQMTLEENGRRTILQLGRG      360
Bd21-3      SRADKLCSRSPFYNNMTKRYELDFRERVGRMGYKVQTSVKNFQMTLEENGRQTILQLGRG      360
Koz-3       SRADKLCSRSPFYNNMTKRYELDFRERVGRMGYKVQTSVKNFQMTLEENGRQTILQLGRV      360
Bd2-3       SRADKLCSRSPFYNNMTKRYELDFRERVGRMGYKVQTSVKNFQMTLEENGRQTILQLGRV      360
Bd21        SRADKLCSRSPFYNNMTKRYELDFRERVGRMGYKVQTSVKNFQMTLEENGRQTILQLGRV      360
ABR6        SRADKLCSRSPFYNNMTKRYELDFRERVGRMGYKVQTSVKNFQMTLEENGRQTILQLGRV      360
RON2        SRADKLCSRSPFYNNMTKRYELDFRERVGRMGYKVQTSVKNFQMTLEENGRQTILQLGRV      360
Bd29-1      SRADKLCSRSPFYNNMTKRYELDFRERVGRMGYKVQTSVKNFQMTLEENGRRTILQLGRV      360
            ***************************************************:*******

Bd30-1      GKSKYIMDFRYPLTGYQAFCICLASMDSKLCCTL  394
Bd3-1       GKSKYIMDFRYPLTGYQAFCICLASMDSKLCCTL  263
Bd1-1       GKSKYIMDFRYPLTGYQAFCICLASMDSKLCCTL  394
Tek-4       GKSKYIMDFRYPLTGYQAFCICLASMDSKLCCTL  394
Bd21-3      GKSKYIMDFRYPLTG-------------------  375
Koz-3       GKSKYIMDFRYPLTGYQAFCICLASMDSKLCCTL  394
Bd2-3       GKSKYIMDFRYPLTGYQAFCICLASMDSKLCCTL  394
Bd21        GKSKYIMDFRYPLTGYQAFCICLASMDSKLCCTL  394
ABR6        GKSKYIMDFRYPLTGYQAFCICLASMDSKLCCTL  394
RON2        GKSKYIMDFRYPLTGYQAFCICLASMDSKLCCTL  394
Bd29-1      GKSKYIMDFRYPLTGYQAFCICLASMDSKLCCTL  394
            ***************
```

BdTLP14

```
Bd30-1     MSLRSMIREMKGEVGAMSLRGLRSRPS-----SG---RDRRVAPDEAARQSSCWAQLPPE     52
Bd3-1      MSFRSLIQEMRDEFGSMSISRHGLRSSRSHRGAGHTPRASAVGP-AEALQQSCWTRLPPE     59
Bd21       MSFRSLIQEMRDEFGSMSISRHGLRSSRSHRGAGHTPRASAVGP-AEALQQSCWTRLPPE     59
Tek-4      MSFRSLIQEMRDEFGSMSISRHGLRSSRSHRGAGHTPRASAVGP-AEALQQSCWTRLPPE     59
ABR6       MSFRSLIQEMRDEFGSMSISRHGLRSSRSHRGAGHTPRASAVGP-AEALQQSCWTRLPPE     59
Bd29-1     MSFRSLIQEMRDEFGSMSISRHGLRSSRSHRGAGHTPRASAVGP-AEALQQSCWTRLPPE     59
Bd2-3      MSFRSLIQEMRDEFGSMSISRHGLRSSRSHRGAGHTPRASAVGP-AEALQQSCWTRLPPE     59
RON2       MSFRSLIQEMRDEFGSMSISRHGLRSSRSHRGAGHTPRASAVGP-AEALQQSCWTRLPPE     59
Koz-3      MSFRSLIQEMRDEFGSMSISRHGLRSSRSHRGAGHTPRASAVGP-AEALQQSCWTRLPPE     59
Bd21-3     MSFRSLIQEMRDEFGSMSISRHGLRSSRSHRGAGHTPRASAVGP-AEALQQSCWTRLPPE     59
Bd1-1      MSFRSLIQEMRDEFGSMSISRHGLRSSRSHRGAGHPPRASAGGP-AEALQQSCWTRLPPE     59
           **:**:*:**:.*.*:**:     * *     :*   *    .*   * *.***::****

Bd30-1     LLREVLGRVEESEPRWPRRRDVVACAGVCQSWRGAVREIVRVPEESGKLTFPISLKQPGP     112
Bd3-1      LLREVLVKIEDSEGCWPSRRDVVSCAGVCRTWRGIMKEVVRVPEASGKLTFPISLKQPGP     119
Bd21       LLREVLVKIEDSEGCWPSRRDVVSCAGVCRTWRGIMKEVVRVPEASGKLTFPISLKQPGP     119
Tek-4      LLREVLVKIEDSEGCWPSRRDVVSCAGVCRTWRGIMKEVVRVPEASGKLTFPISLKQPGP     119
ABR6       LLREVLVKIEDSEGCWPSRRDVVSCAGVCRTWRGIMKEVVRVPEASGKLTFPISLKQPGP     119
Bd29-1     LLREVLVKIEDSEGCWPSRRDVVSCAGVCRTWRGIMKEVVRVPEASGKLTFPISLKQPGP     119
Bd2-3      LLREVLVKIEDSEGCWPSRRDVVSCAGVCRTWRGIMKEVVRVPEASGKLTFPISLKQPGP     119
RON2       LLREVLVKIEDSEGCWPSRRDVVSCAGVCRTWRGIMKEVVRVPEASGKLTFPISLKQPGP     119
Koz-3      LLREVLVKIEDSEGCWPSRRDVVSCAGVCRTWRGIMKEVVRVPEASGKLTFPISLKQPGP     119
Bd21-3     LLREVLVKIEDSEGCWPSRRDVVSCAGVCRTWRGIMKEVVRVPEASGKLTFPISLKQPGP     119
Bd1-1      LLREGLGKIEDSEGCWPSRRDVVSCAGVCRTWRGIMKEVVRVPEASGKLTFPISLKQPGP     119
           **** * ::*:**  ** *****:*****::*** ::*:***** ***************

Bd30-1     RDAPIKCFIRRNRATHTYCLYIGLTDALADDGKFLLAARKYHRPTCTEYLILLDRTDMSK     172
Bd3-1      KDGTLRCFIRRNRTTQTYSLYIGLTEALADDGKFLLAARKCRKATYTDYLISLDMGDMSK     179
Bd21       KDGTLRCFIRRNRTTQTYSLYIGLTEALADDGKFLLAARKCRKATYTDYLISLDMGDMSK     179
Tek-4      KDGTLRCFIRRNRTTQTYSLYIGLTEALADDGKFLLAARKCRKATYTDYLISLDMGDMSK     179
ABR6       KDGTLRCFIRRNRTTQTYSLYIGLTEALADDGKFLLAARKCRKATYTDYLISLDMGDMSK     179
Bd29-1     KDGTLRCFIRRNRTTQTYSLYIGLTEALADDGKFLLAARKCRKATYTDYLISLDMGDMSK     179
Bd2-3      KDGTLRCFIRRNRTTQTYSLYIGLTEALADDGKFLLAARKCRKATYTDYLISLDMGDMSK     179
RON2       KDGTLRCFIRRNRTTQTYSLYIGLTEALADDGKFLLAARKCRKATYTDYLISLDMGDMSK     179
Koz-3      KDGTLRCFIRRNRTTQTYSLYIGLTEALADDGKFLLAARKCRKATYTDYLISLDMGDMSK     179
Bd21-3     KDGTLRCFIRRNRTTQTYSLYIGLTEALADDGKFLLAARKCRKATYTDYLISLDMGDMSK     179
Bd1-1      KDGTLRCFIRRNRTTQTYSLYIGLTEALADDGKFLLAARKCRKATYTDYLISLDMGDMSK     179
           :*. ::*******:*:**.******:************** :: * *:*** **  ****

Bd30-1     GSQTCIGKLRSNFLRTKFTVYDAHPPHSGAVVSK--------------RWSSGNYPVSHI     218
Bd3-1      GSSTYIGKLRSNFLGTKFTVYDAHPPYDGAVVSKSRSARVIGLNRVSPRVPAGNYPVSHI     239
Bd21       GSSTYIGKLRSNFLGTKFTVYDAHPPYDGAVVSKSRSARVIGLNRVSPRVPAGNYPVSHI     239
Tek-4      GSSTYIGKLRSNFLGTKFTVYDAHPPYDGAVVSKSRSARVIGLNRVSPRVPAGNYPVSHI     239
ABR6       GSSTYIGKLRSNFLGTKFTVYDAHPPYDGAVVSKSRSARVIGLNRVSPRVPAGNYPVSHI     239
Bd29-1     GSSTYIGKLRSNFLGTKFTVYDAHPPYDGAVVSKSRSARVIGLNRVSPRVPAGNYPVSHI     239
Bd2-3      GSSTYIGKLRSNFLGTKFTVYDAHPPYDGAVVSKSRSARVIGLNRVSPRVPAGNYPVSHI     239
RON2       GSSTYIGKLRSNFLGTKFTVYDAHPPYDGAVVSKSRSARVIGLNRVSPRVPAGNYPVSHI     239
Koz-3      GSSTYIGKLRSNFLGTKFTVYDAHPPYDGAVVSKSRSARVIGLNRVSPRVPAGNYPVSHI     239
Bd21-3     GSSTYIGKLRSNFLGTKFTVYDAHPPYDGAVVSKSRSARVIGLNRVSPRVPAGNYPVSHI     239
Bd1-1      GSSTYIGKLRSNFLGTKFTVYDAHPPYDGAVVSKSRSARVIGLNRVSPRVPAGNYPVSHI     239
           **.* ********* ***********:.******              *  :********
```

```
Bd30-1      YYDVNVLGSRGPRRMRCVMDSIPVSAI-KGGTAPTQTGFPSSSHDSFASIPFFKSKSSRR      277
Bd3-1       SYELNVLGSRGPRRMNCVMNSIPASAVEEGGKAPTQTEFPRSNLDSFPSIPFFRSKSARI      299
Bd21        SYELNVLGSRGPRRMNCVMNSIPASAVEEGGKAPTQTEFPRSNLDSFPSIPFFRSKSARI      299
Tek-4       SYELNVLGSRGPRRMNCVMNSIPASAVEEGGKAPTQTEFPRSNLDSFPSIPFFRSKSARI      299
ABR6        SYELNVLGSRGPRRMNCVMNSIPASAVEEGGKAPTQTEFPRSNLDSFPSIPFFRSKSARI      299
Bd29-1      SYELNVLGSRGPRRMNCVMNSIPASAVEEGGKAPTQTEFPRSNLDSFPSIPFFRSKSARI      299
Bd2-3       SYELNVLGSRGPRRMNCVMNSIPASAVEEGGKAPTQTEFPRSNLDSFPSIPFFRSKSARI      299
RON2        SYELNVLGSRGPRRMNCVMNSIPASAVEEGGKAPTQTEFPRSNLDSFPSIPFFRSKSARI      299
Koz-3       SYELNVLGSRGPRRMNCVMNSIPASAVEEGGKAPTQTEFPRSNLDSFPSIPFFRSKSARI      299
Bd21-3      SYELNVLGSRGPRRMNCVMNSIPASAVEEGGKAPTQTEFPRSNLDSFPSIPFFRSKSARI      299
Bd1-1       SYELNVLGSRGPRRMNCVMNSIPASAVEEGGKAPTQTEFPRSNLDSFPSIPFFRSKSARI      299
             *::***********.***:***.**: :**.***** ** *. *** *****:***:* 

Bd30-1      DSSTTQLSTQEESKLVLKNKSPRWQEHLQCWCLNFHGRVTVASVKNFQLVPSDESAPTNH      337
Bd3-1       DSTASQLPTQMEEKLVLKNKSPRWHEQLQCWCLNFRGRVTVASVKNFQLVASDDNGPANQ      359
Bd21        DSTASQLPTQMEEKLVLKNKSPRWHEQLQCWCLNFRGRVTVASVKNFQLVASDDNGPANQ      359
Tek-4       DSTASQLPTQMEEKLVLKNKSPRWHEQLQCWCLNFRGRVTVASVKNFQLVASDDNGPANQ      359
ABR6        DSTASQLPTQMEEKLVLKNKSPRWHEQLQCWCLNFRGRVTVASVKNFQLVASDDNGPANQ      359
Bd29-1      DSTASQLPTQMEEKLVLKNKSPRWHEQLQCWCLNFRGRVTVASVKNFQLVASDDNGPANQ      359
Bd2-3       DSTASQLPTQMEEKLVLKNKSPRWHEQLQCWCLNFRGRVTVASVKNFQLVASDDNGPANQ      359
RON2        DSTASQLPTQMEEKLVLKNKSPRWHEQLQCWCLNFRGRVTVASVKNFQLVASDDNGPANQ      359
Koz-3       DSTASQLPTQMEEKLVLKNKSPRWHEQLQCWCLNFRGRVTVASVKNFQLVASDDNGPANQ      359
Bd21-3      DSTASQLPTQMEEKLVLKNKSPRWHEQLQCWCLNFRGRVTVASVKNFQLVASDDNGPANQ      359
Bd1-1       DSTASQLPTQMEEKLVLKNKSPRWHEQLQCWCLNFRGRVTVASVKNFQLVASDDNGPANQ      359
            **:::** ** *.***********:*:********:************** **:..*:*:

Bd30-1      ELDDATLQFGKIGKDMFSMDYRYPLSAFQAFAICLSSFDTKIACE      382
Bd3-1       EHDKVILQFGKIGKDLFTMDYRYPISAFQSFAICLSSFDTKIACE      404
Bd21        EHDKVILQFGKIGKDLFTMDYRYPISAFQSFAICLSSFDTKIACE      404
Tek-4       EHDKVILQFGKIGKDLFTMDYRYPISAFQSFAICLSSFDTKIACE      404
ABR6        EHDKVILQFGKIGKDLFTMDYRYPISAFQSFAICLSSFDTKIACE      404
Bd29-1      EHDKVILQFGKIGKDLFTMDYRYPISAFQSFAICLSSFDTKIACE      404
Bd2-3       EHDKVILQFGKIGKDLFTMDYRYPISAFQSFAICLSSFDTKIACE      404
RON2        EHDKVILQFGKIGKDLFTMDYRYPISAFQSFAICLSSFDTKIACE      404
Koz-3       EHDKVILQFGKIGKDLFTMDYRYPISAFQSFAICLSSFDTKIACE      404
Bd21-3      EHDKVILQFGKIGKDLFTMDYRYPISAFQSFAICLSSFDTKIACE      404
Bd1-1       EHDKVILQFGKIGKDLFTMDYRYPISAFQSFAICLSSFDTKIACE      404
            * *.. *********:*:******:****:***************
```
