# Supplementary material for: Comprehensive Genome-Wide Natural Variation and Expression Analysis of Tubby-like Proteins Gene Family in Brachypodium distachyon
Source: Plants (Basel). 2024 Mar 29;13(7):987. doi: 10.3390/plants13070987 (PMC11013449; doi:10.3390/plants13070987)
Supplement: Supplementary file 1 [file plants-13-00987-s001.zip › Figure S8. 3D structures with residues.pdf]

Supplementary Figure S8. Wild type and mutated amino acid interactions in TLP1, 3, 8 and 13 predicted by DDmut [55]. Wild type amino acid interactions are shown on the left side, and mutated amino acid interactions on the right side.

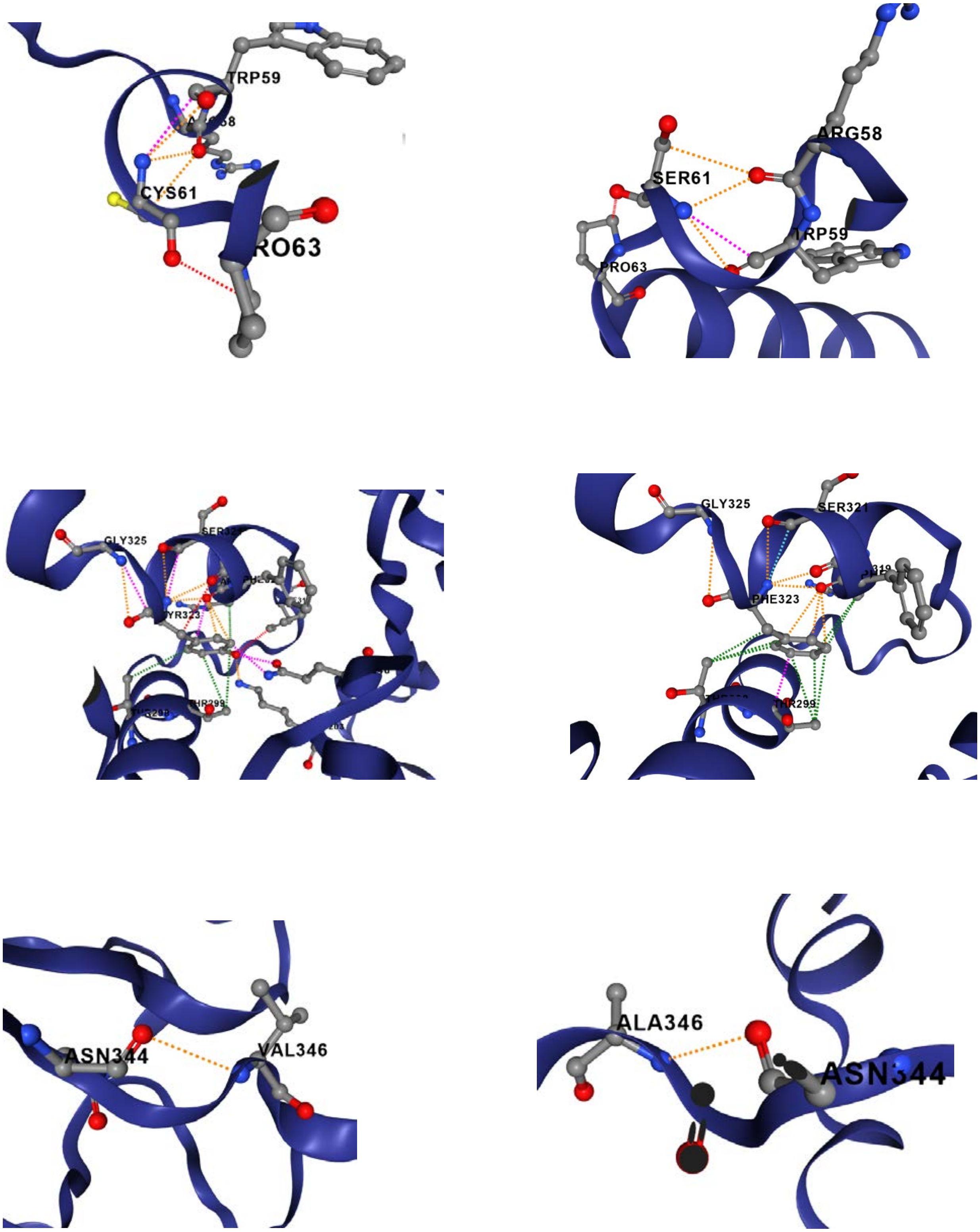

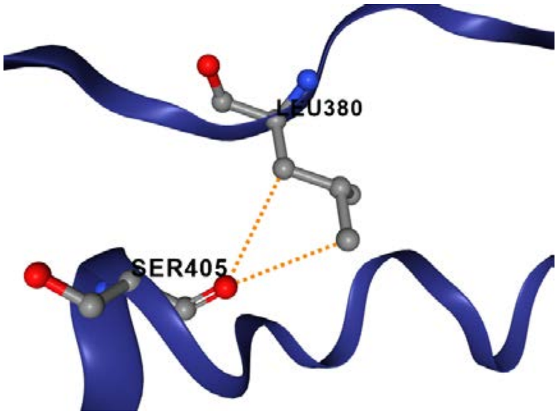
LEU380
SER405

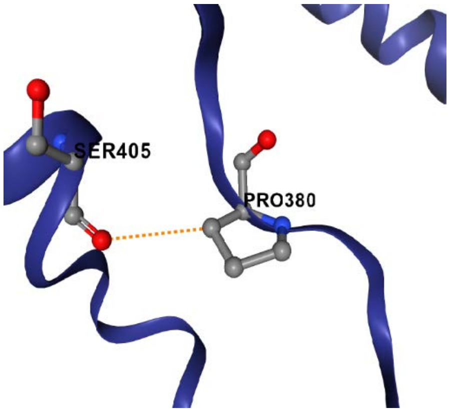
SER405
PRO380

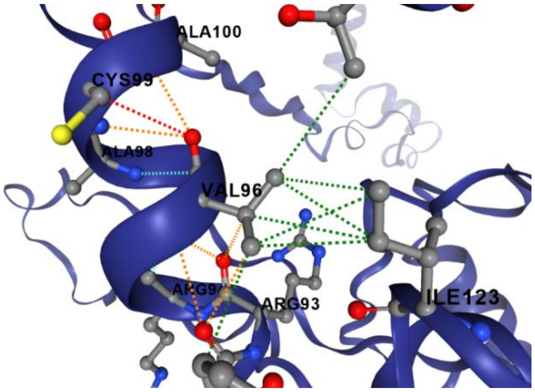
ALA100
CYS99
ALA98
VAL96
ARG93
ILE123

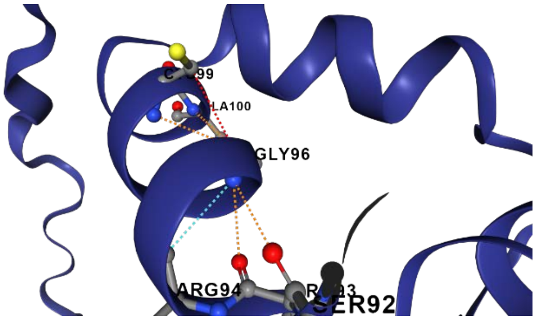
ALA100
GLY96
ARG94
SER92

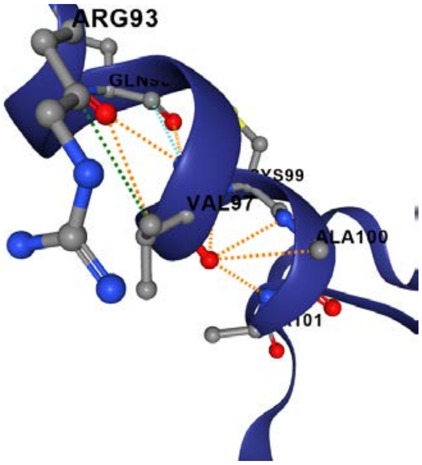
ARG93
VAL97
ALA100

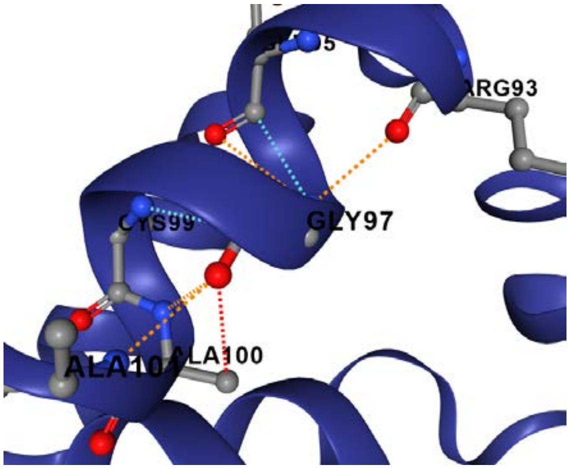
RG93
GLY97
CYS99
ALA101
ALA100

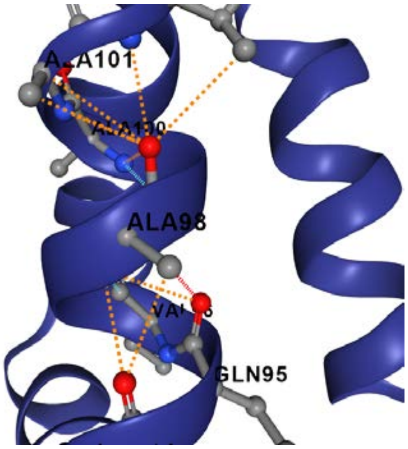
ALA98
GLN95

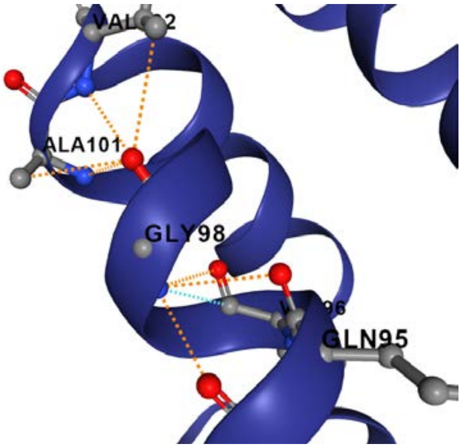
ALA101
GLY98
GLN95

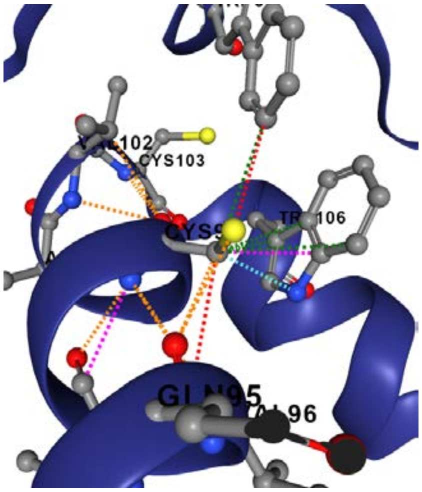
CYS103

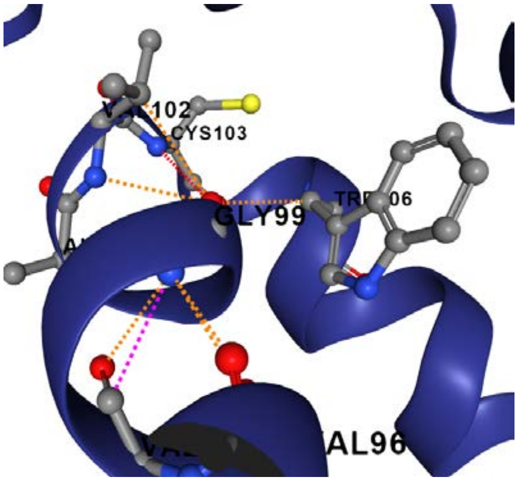
CYS103
GLY99

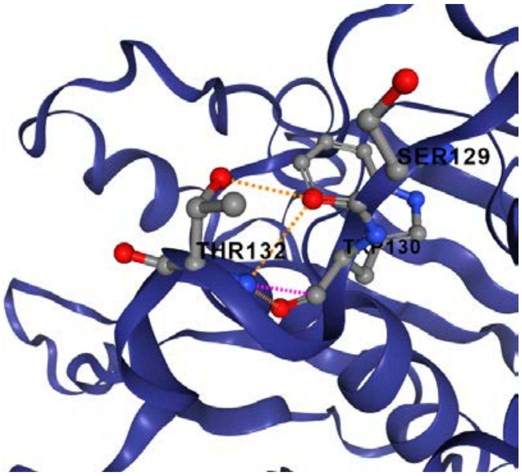
SER129
THR132

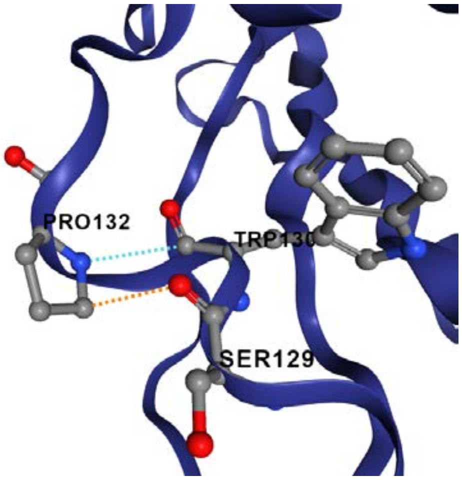
PRO132
TRP130
SER129

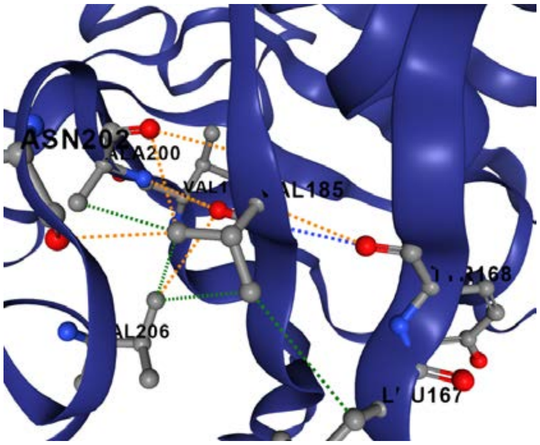
ASN202
ALA200

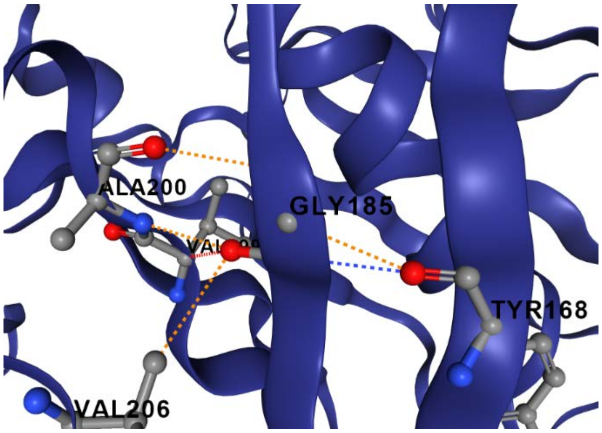
ALA200
GLY185
TYR168
VAL206

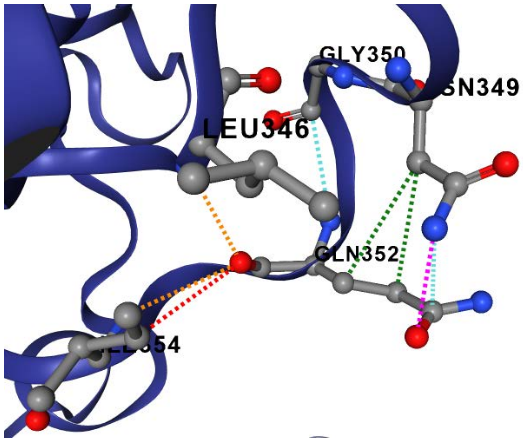
GLY350
LEU346
GLN352

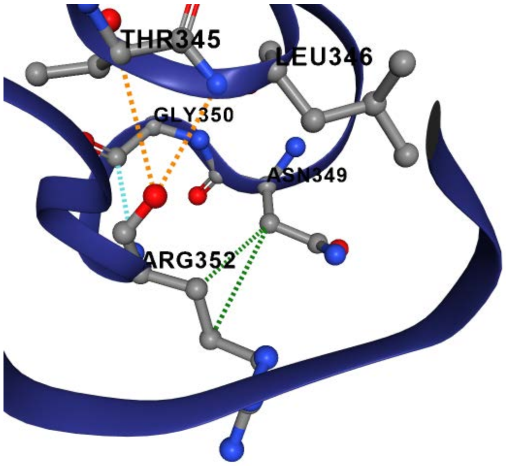
THR345
LEU346
GLY350
ASN349
ARG352
