# Supplementary material for: Comprehensive Genome-Wide Natural Variation and Expression Analysis of Tubby-like Proteins Gene Family in Brachypodium distachyon
Source: Plants (Basel). 2024 Mar 29;13(7):987. doi: 10.3390/plants13070987 (PMC11013449; doi:10.3390/plants13070987)

Supplementary Figure S1. BdTLPs protein domains

Bradi2g24110   F-box 59-106 TUBBY 120-445 TLP1 I1HJ14

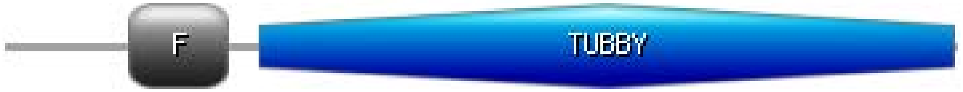

Bradi3g13080   F-box 61-106 TUBBY 120-447 TLP2 I1I0A3

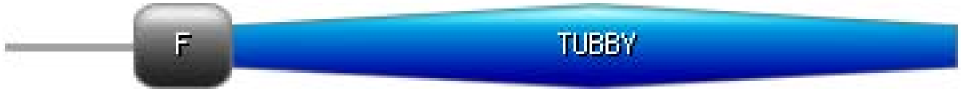

Bradi5g27110 F-box 59-108 TUBBY 120-455 TLP3 I1J3P3

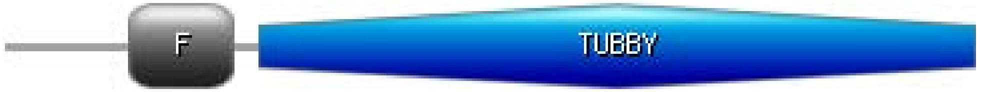

Bradi4g41820 F-box 53-101 TUBBY 114-439 TLP4 I1IU46

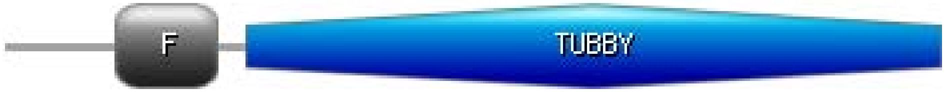

Bradi2g56070 F-box 59-107 TUBBY 120-448 TLP5 I1HTR2

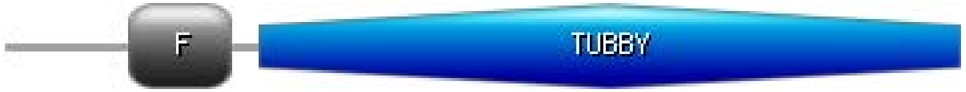

Bradi4g24740 F-box 53-101 TUBBY 114-445 TLP6 I1IN97

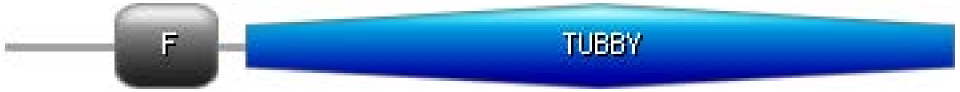

Bradi1g18380 F-box 46-94 TUBBY 107-382 TLP7 I1GRC6

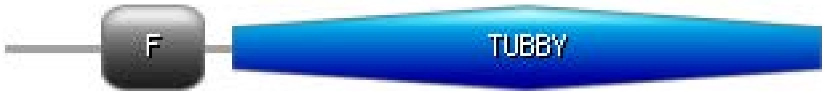

Bradi3g52767 F-box 68-114 TUBBY 129-428 TLP8 I1ID09

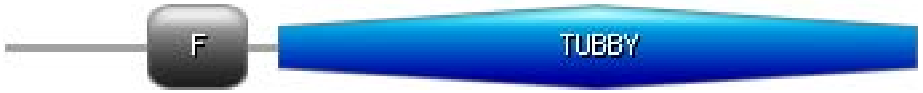

Bradi2g46350 F-box 35-70 TUBBY 100-360 TLP9 I1HQB2

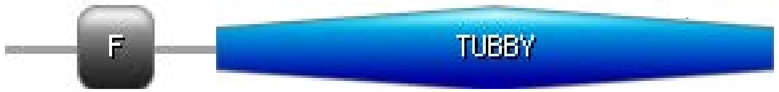

Bradi2g19960 F-box 54-96 TUBBY 115-375 TLP10 I1HHN7

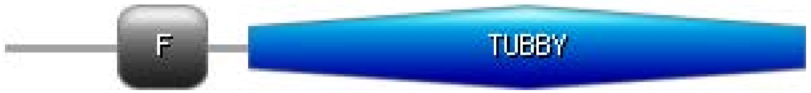

Bradi3g05930   TUBBY 140-393 TLP13 A0A0Q3J640

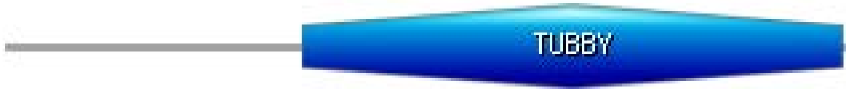

Bradi1g62390 F-box 53-99 TUBBY 114-404 TLP14 I1H5G2

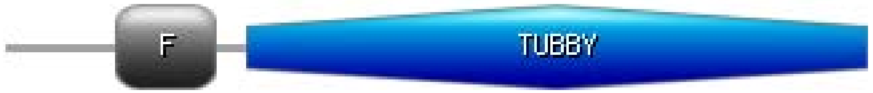

Supplementary Figure S2. AtTLPs protein domains

At1g76900 F-box 57-103 TUBBY 118-455 TLP1 Q9ZP59

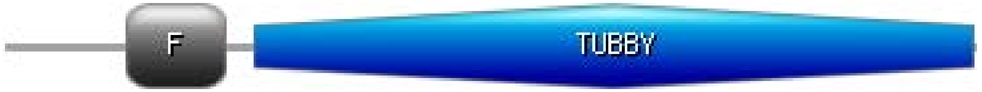

At2g18280 F-box 48-90 TUBBY 109-393 TLP2 Q8GVE5

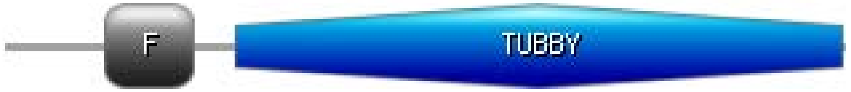

At2g47900 F-box 52-100 TUBBY 113-407 TLP3 Q8VY21

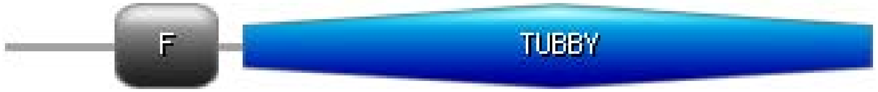

At1g43640 F-box 55-102 TUBBY 116-429 TLP5 Q944S3

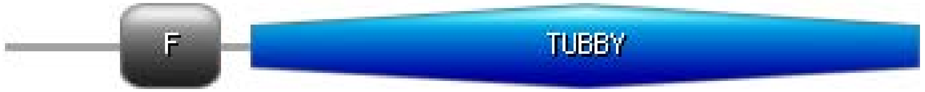

At1g47270 F-box 69-115 TUBBY 130-413 TLP6 Q0WPY0

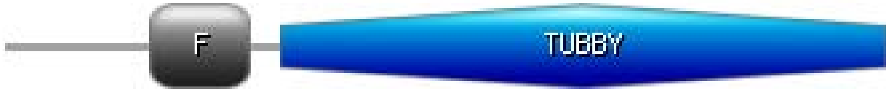

At1g53320 F-box 49-88 TUBBY 105-379 TLP7 Q93VI8

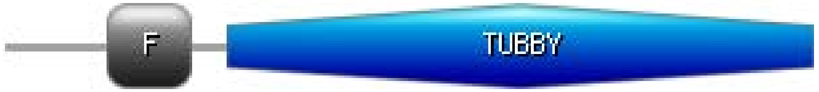

At1g16070   TUBBY 147-392 TLP8 Q9S9M8

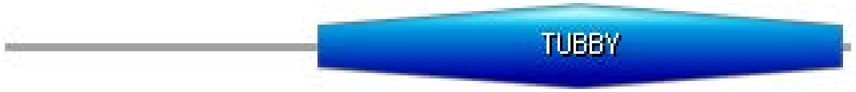

At3g06380   F-box 33-80 TUBBY 95-380 TLP9 Q9SQU1

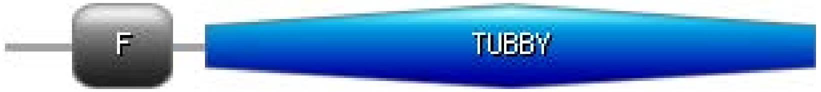

At1g25280 F-box 59-107   TUBBY 120-445 TLP10 Q9FRH7

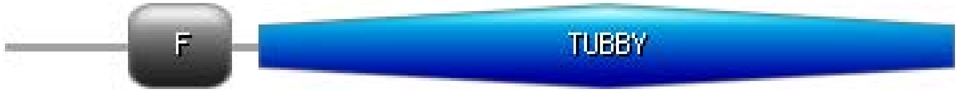

At5g18680   F-box 39-80 TUBBY 101-389 TLP11 Q6NPQ1

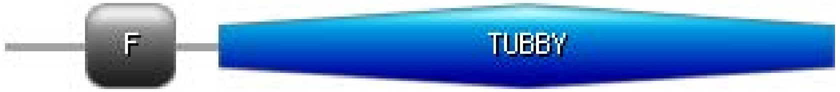

Supplementary Figure S3. OsTLPs protein domains

Os05g0437900 F-box 59-107 TUBBY 120-445 TLP1 Q75HX5

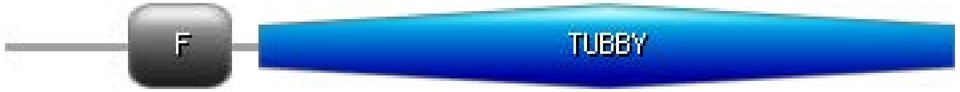

Os08g0103300 F-box 61-106 TUBBY 120-451 TLP2 Q69U54

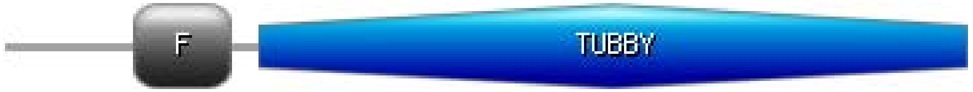

Os04g0687900 F-box 56-105 TUBBY 117-462 TLP3 Q7XSV4

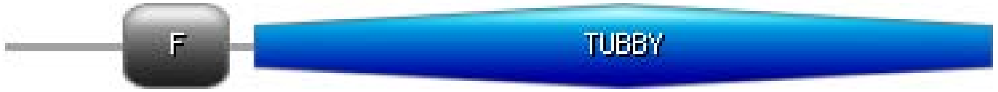

Os12g0163400 F-box 59-107 TUBBY 120-445 TLP4 Q2QXB2

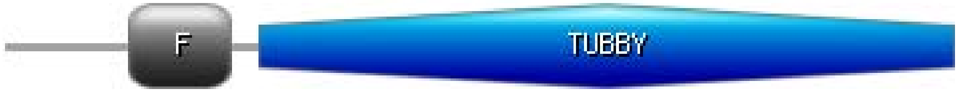

Os01g0866800 F-box 52-100 TUBBY 113-448 TLP5 Q8LJA9

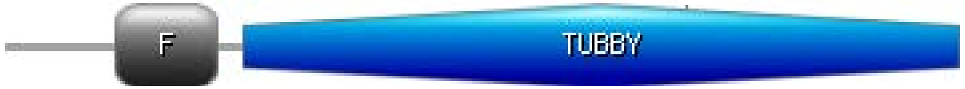

Os11g0163600 F-box 53-101 TUBBY 114-440 TLP6 Q53PP5

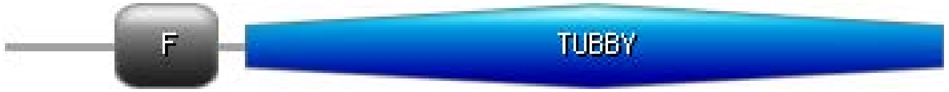

Os07g0667000 F-box 39-80 TUBBY 101-406 TLP7 Q8H485

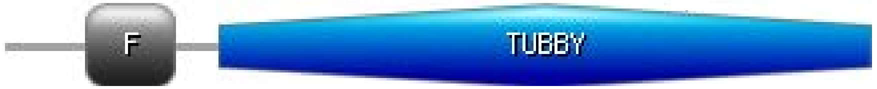

Os02g0705300 F-box 54-111 TUBBY 129-428 TLP8 Q6Z2G9

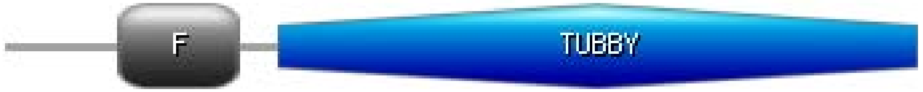

Os01g0674500 F-box 30-65 TUBBY 95-355 TLP9 Q5QM27

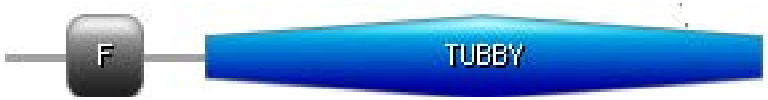

Os05g0514300 F-box 52-94 TUBBY 113-372 TLP10 Q68Y48

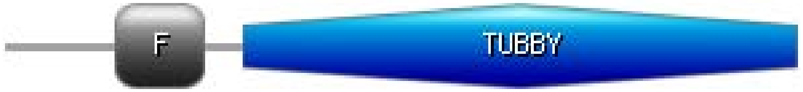

Os05g0560400 F-box 22-78 TUBBY 86-352 TLP11 Q688Y7

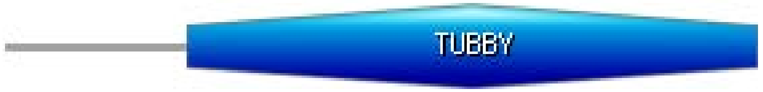

Os01g0759100 F-box 51-92 TUBBY 112-368 TLP12 Q94DT9

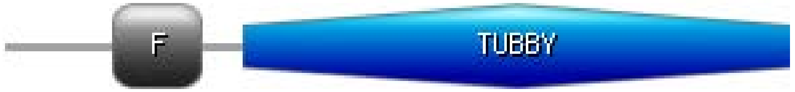

Os02g0179400 TUBBY 161-419 TLP13 Q6ETL2

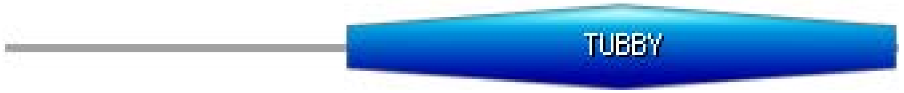

Os03g0351400 F-box 50-100   TUBBY 113-403 TLP14 Q10LG8

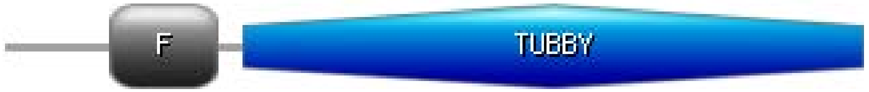

Supplementary Figure S4. SbTLP protein domains

SORBI_3009G152200 F-box 59-107 TUBBY 120-445 TLP1 C5YYQ1

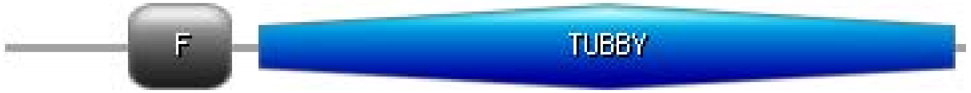

SORBI_3007G002500 F-box 58-104 TUBBY 117-443 TLP2 A0A1B6PER5

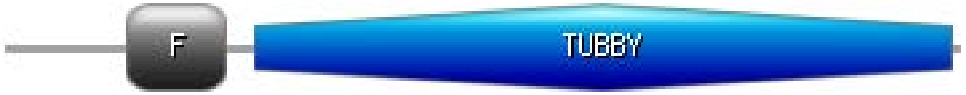

SORBI_3006G277200 F-box 51-98 TUBBY 112-448 TLP3 A0A1Z5RFZ9

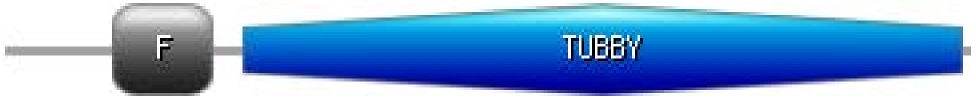

SORBI 3008G047400 F-box 53-101 TUBBY 114-432 TLP4 A0A1B6PBC9

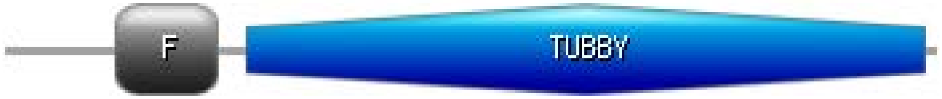

SORBI_3003G368000 F-box 59-107 TUBBY 120-475 TLP5 C5XQM9

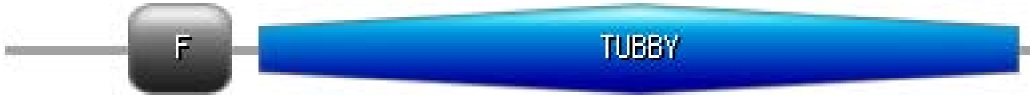

SORBI_3005G047300 F-box 53-99 TUBBY 114-451 TLP6 C5Y569

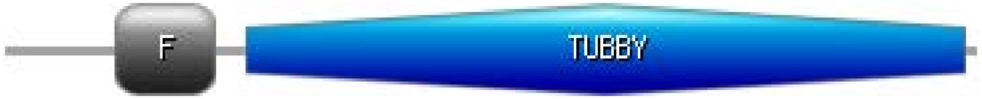

SORBI_3002G408300 F-box 52-99 TUBBY 113-394 TLP7 C5X4V0

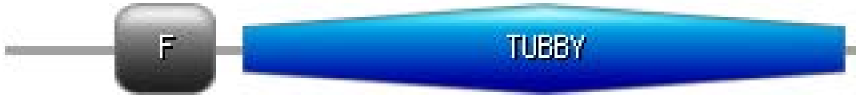

SORBI_3004G267600 F-box 71-117 TUBBY 132-428 TLP8 C5Y0B7

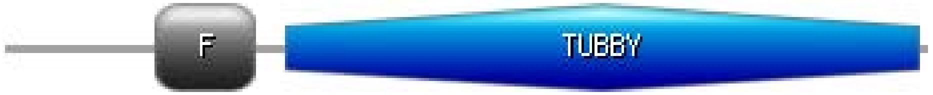

SORBI_3003G253800 F-box 37-92 TUBBY 100-355 TLP9 C5XG39

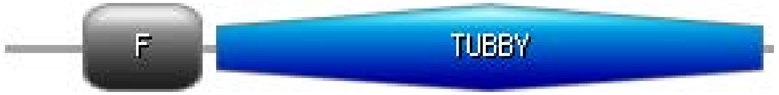

SORBI_3009G195900 F-box 52-94 TUBBY 113-367 TLP10 C5Z0X1

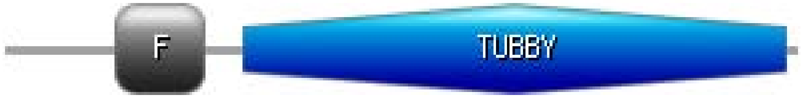

SORBI_3009G230500 F-box 25-76 TUBBY 88-346 TLP11 A0A1Z5R3X4

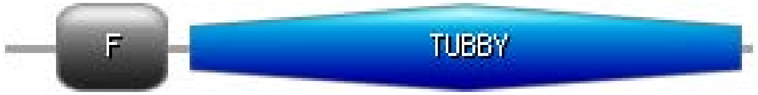

SORBI_3004G064700 TUBBY 169-417 TLP13 A0A194YN41

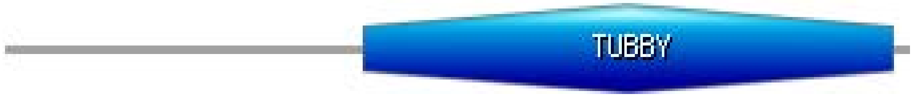

SORBI_3001G372000 F-box 50-91 TUBBY 111-394 TLP14 A0A1B6QN76

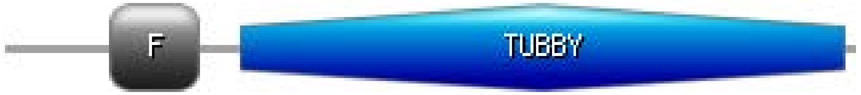

Supplementary Figure S6. BdTLP gene expression from dark to light transition (E-MTAB-6445)

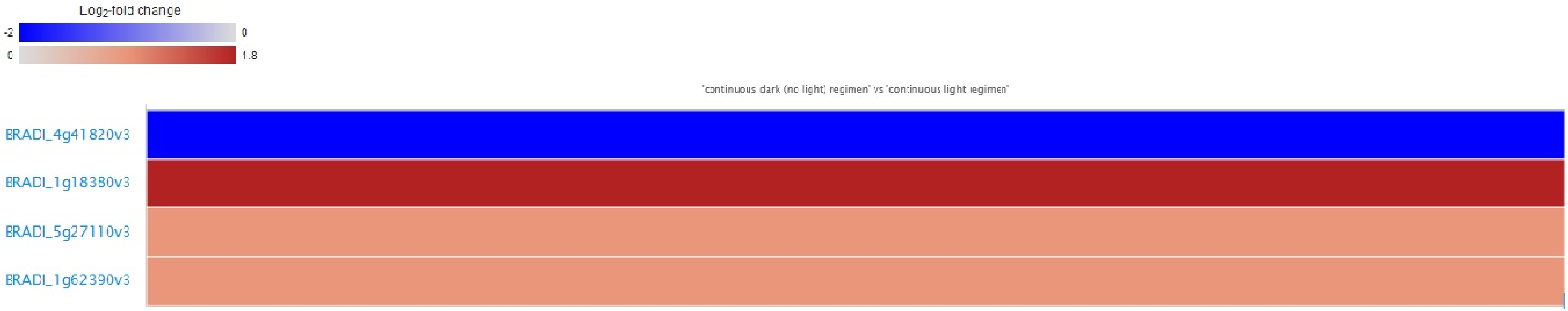

Supplement: Supplementary file 1 [file plants-13-00987-s001.zip › Figures S1-S4, S6.pdf]
